# Supplementary material for: Cold call: the acoustic repertoire of Ross Sea killer whales (Orcinus orca, Type C) in McMurdo Sound, Antarctica
Source: R Soc Open Sci. 2020 Feb 5;7(2):191228. doi: 10.1098/rsos.191228 (PMC7062068; doi:10.1098/rsos.191228)
Supplement: Appendix 1: Table 1 and 2 [file rsos191228supp1.pdf]

Supplementary Material, Appendix 1

Rebecca Wellard[1,2], Robert L. Pitman[3,4], John Durban[5], Christine Erbe[1] (2020).
Cold Call: The Acoustic Repertoire of Ross Sea Killer Whales (*Orcinus orca*, Type C) in McMurdo Sound, Antarctica. *Royal Society Open Science.*

[1] Centre for Marine Science & Technology, Curtin University, GPO Box U1987, Perth, WA 6845, Australia.

[2] Project ORCA, Perth, WA 6026, Australia.

[3] Antarctic Ecosystem Research Division, Southwest Fisheries Science Center, National Marine Fisheries Service, 8901 La Jolla Shores Dr., La Jolla, CA 92037, USA.

[4] Marine Mammal Institute, Oregon State University, 2030 SE Marine Science Drive, Newport, OR 97365, USA.

[5] Marine Mammal and Turtle Division, Southwest Fisheries Science Center, National Marine Fisheries Service, 8901 La Jolla Shores Dr., La Jolla, CA 92037, USA.

This Supplementary Material, Appendix 1 contains the following:

- **Table 1.** *List of parameters measured to quantify the spectro-temporal structure of call types recorded from Type C killer whales in McMurdo Sound, Antarctica. (V): measured visually from spectrograms in Raven; (R): computed by Raven.*

- **Table 2.** *Descriptive statistics (mean ±SD) of catalogued and categorised Type C killer whale calls from McMurdo Sound, Ross Sea. Here we present measurements of the fundamental frequency for whistle (W) and biphonic whistle (Bi) components and measurements of the entire component for burst-pulse sounds (P). Measurements of individual components are displayed here, along with measurements of the entire call when multiple components were present. Spectrograms of each call type are presented in the Supplementary Material, Appendix 2, call catalogue.*

Table 1. List of parameters measured to quantify the spectro-temporal structure of call types recorded from Type C killer whales in McMurdo Sound, Antarctica. (V): measured visually from spectrograms in Raven; (R): computed by Raven.

Parameter	Abbreviation	Description
**Duration (R)**	Dur	Time duration [s] of the entire call
**Duration 90% (R)**	Dur90%	Useful for burst-pulse sounds and whistles, the duration [s] containing 90% of the call energy
**Minimum Frequency (V)**	Fmin	Lowest frequency [Hz] of the call in the case of burst-pulse sounds and lowest frequency of the fundamental contour in the case of whistles
**Maximum Frequency (V)**	Fmax	Highest frequency [Hz] of the call in the case of burst-pulse sounds and highest frequency of the fundamental contour in the case of whistles
**Start Frequency (V)**	Fstart	Useful for whistles, the frequency [Hz] at the start of the fundamental contour
**End Frequency (V)**	Fend	Useful for whistles, the frequency [Hz] at the end of the fundamental contour
**Delta Frequency (R)**	Fdelta	Range of frequencies spanned by the burst-pulse sound or the fundamental whistle contour (Fdelta = Fmax - Fmin)
**Bandwidth 90% (R)**	BW90%	Useful for burst-pulse sounds, the bandwidth [Hz] containing 90% of the call energy (i.e., difference between the frequencies at the 5th and 95th energy percentiles)
**Peak Frequency (R)**	Fpeak	Useful for burst-pulse sounds, the frequency [Hz] at which the call spectrum has its maximum energy
**Centre Frequency (R)**	Fcentre	Useful for burst-pulse sounds, the frequency [Hz] that divides the call spectrum into two frequency bands of equal energy
**1st Quartile Frequency (R)**	Q1F	Useful for burst-pulse sounds, the frequency [Hz] that divides the call spectrum into two frequency bands containing 25% and 75% of the energy in the call
**3rd Quartile Frequency (R)**	Q3F	Useful for burst-pulse sounds, the frequency [Hz] that divides the call spectrum into two frequency bands containing 75% and 25% of the energy in the call
**Minimum Entropy (R)**	MinEnt	Useful for burst-pulse sounds, the minimum entropy over all time bins in the call spectrogram [bits]
**Maximum Entropy (R)**	MaxEnt	Useful for burst-pulse sounds, the maximum entropy over all time bins in the call spectrogram [bits]
**Average Entropy (R)**	AvgEnt	Useful for burst-pulse sounds, the average entropy over all time bins in the call spectrogram [bits]
**Number of Extrema (V)**	Ext	Extrema are local maxima and minima in the whistle contour, i.e., where the first derivative of the whistle contour with respect to time is zero
**Inflection points (V)**	Infl	At inflection points, the curvature of the whistle contour changes from clockwise to counter-clockwise or vice versa. The second derivative of the whistle contour with respect to time is zero.
**FM rate (V)**	FM	The ratio of the number of inflection points and duration [1/s]
**Number of Steps (V)**	Steps	A discontinuity in the whistle contour, where the contour makes a jump in frequency without any gap in time
**Harmonics (V)**	Harm	The presence of harmonics in whistles was noted as a binary response (y/n)

Table 2. Descriptive statistics (mean ±SD) of catalogued and categorised Type C killer whale calls from McMurdo Sound, Ross Sea. Here we present measurements of the fundamental frequency for whistle (W) and biphonic whistle (Bi) components and measurements of the entire component for burst-pulse sounds (P). Measurements of individual components are displayed here, along with measurements of the entire call when multiple components were present. Spectrograms of each call type are presented in the Supplementary Material, Appendix 2, call catalogue.

Call Type	Component Type	Component Number	Statistic	Dur [s]	Fmin [Hz]	Fmax [Hz]	Fdelta [Hz]	BW 90% [Hz]	Fpeak [Hz]	Fcentre [Hz]	Q1F [Hz]	Q3F [Hz]	MinEnt [bits]	MaxEnt [bits]	AvgEnt [bits]	Dur90% [s]	Fstart [Hz]	Fend [Hz]	Ext	Infl	FM	Steps
**McM1**	**P**	**1**	**Mean**	0.21	1007	12988	11981	3612	2791	3021	2588	3790	3.4	6.8	5.0	0.16						
n=101			***SD***	0.09	478	5032	4997	1790	623	858	396	1301	0.5	0.4	0.5	0.06						
	**P**	**2**	**Mean**	0.18	830	31350	30521	9743	4073	4402	2951	6354	5.1	7.2	6.2	0.14						
			SD	0.03	367	14593	14686	10008	4041	2185	1190	4682	0.7	0.9	0.8	0.05						
	**P**	**3**	**Mean**	0.85	884	14401	13517	4502	4155	4071	3012	4852	3.6	6.5	4.3	0.54						
			SD	0.23	321	5315	5407	1840	1140	904	794	880	0.9	0.5	0.4	0.12						
	**Bi**	**4**	**Mean**	0.60	7284	12809	5525	3054	8536	8798	8161	9642	2.8	6.0	3.7	0.44	12492	8893	3	3	5.3	0
			SD	0.12	1289	1084	1181	1228	1893	1597	1572	1556	0.7	0.5	0.5	0.10	1826	1746	3	3	5.3	0
		**Entire**	**Mean**	1.25	752	30358	29606	5805	4153	4048	2979	4802	3.3	6.9	4.8	0.76						
			SD	0.30	396	13139	13327	3194	1143	952	779	979	0.7	0.8	0.3	0.17						
**McM1a**	**P**	**1**	**Mean**	0.22	1634	43989	42355	20361	3188	6352	3609	8824	5.1	8.5	7.2	0.19						
n=40			***SD***	0.04	304	9059	9170	11167	978	4395	1735	4816	1.2	0.6	0.7	0.04						
	**P**	**2**	**Mean**	0.78	1061	14350	13289	6469	4881	4881	3621	5414	4.1	6.8	4.6	0.60						
			SD	0.14	477	1768	2002	1756	1719	1317	569	1474	0.8	0.4	0.4	0.12						
	**Bi**	**3**	**Mean**	0.68	7950	13116	5166	2953	9211	9398	8818	10184	2.8	6.0	3.7	0.54	13116	9390	4	4	5.3	0
			SD	0.12	799	1013	934	630	1271	1045	973	898	0.8	0.4	0.3	0.17	1013	1110	3	2	3.5	0
		**Entire**	**Mean**	0.94	907	43937	43030	14133	4881	4922	3650	6768	3.7	7.2	5.3	0.73						
			SD	0.12	615	8997	9387	9608	1719	1318	711	3702	0.8	0.9	0.6	0.10						
**McM2**	**P**	**1**	**Mean**	0.13	1162	14684	13522	3788	3089	3110	2510	3895	4.0	6.7	5.4	0.10						
n=111			***SD***	0.09	472	8727	8833	1826	1098	865	636	1005	0.8	0.9	0.5	0.06						
	**P**	**2**	**Mean**	0.82	915	16564	15648	5836	3750	3787	3199	4695	3.5	6.7	4.3	0.58						
			SD	0.17	330	8864	8921	2048	1197	1049	953	931	0.8	0.7	0.4	0.12						
	**Bi**	**3**	**Mean**	0.70	7778	12064	4285	2688	9504	9581	8817	10273	2.5	5.3	3.3	0.51	10227	9207	3	4	5.6	0
			SD	0.14	1226	1545	1195	830	1693	1406	1357	1357	0.6	0.5	0.6	0.13	2579	1591	2	3	4.2	0
		**Entire**	**Mean**	0.88	986	16115	14924	5878	3766	3769	3183	4685	3.2	6.0	4.2	0.61						
			SD	0.18	420	8783	8178	2006	1211	1047	954	926	0.7	0.6	0.5	0.13						

Call Type	Component Type	Component Number	Statistic	Dur [s]	Fmin [Hz]	Fmax [Hz]	Fdelta [Hz]	BW 90% [Hz]	Fpeak [Hz]	Fcentre [Hz]	Q1F [Hz]	Q3F [Hz]	MinEnt [bits]	MaxEnt [bits]	AvgEnt [bits]	Dur90% [s]	Fstart [Hz]	Fend [Hz]	Ext	Infl	FM	Steps
**McM3**	**P**	**1**	**Mean**	0.84	981	17114	16133	7341	3996	4369	3300	6534	3.9	7.2	4.9	0.64						
n=130			***SD***	0.19	376	7939	7963	4150	1857	1913	962	2677	0.9	0.8	0.6	0.14						
	**Bi**	**2**	**Mean**	0.61	3628	10666	7037	4686	5661	6120	5248	7511	3.1	5.9	4.1	0.50	4020	7989	2	3	4.8	1
			SD	0.18	1634	1524	1852	2016	2247	2393	1963	2421	0.6	0.7	0.4	0.18	1569	2883	1	1	2.2	1
		**Entire**	**Mean**	0.86	987	17328	16341	7425	3984	4373	3308	6533	3.8	7.2	5.0	0.65						
			SD	0.18	388	7674	7723	4044	1867	1911	973	2657	0.9	0.7	0.5	0.14						
**McM3a**	**P**	**1**	**Mean**	0.98	681	17377	16696	4297	2398	2961	2375	4586	2.9	7.0	4.4	0.45						
n=30			***SD***	0.10	182	5073	5180	2200	104	1052	125	1933	0.7	0.8	0.3	0.14						
	**Bi**	**2**	**Mean**	0.45	3178	9832	6654	3742	4250	4898	4102	5656	2.7	6.0	3.6	0.28	3030	7461	2	3	6.8	2
			SD	0.11	1094	1028	1906	2284	1719	1374	1330	1276	0.7	0.3	0.6	0.17	1571	3939	1	1	2.5	1
		**Entire**	**Mean**	0.98	612	17316	16704	4297	2398	2961	2375	4586	2.9	7.0	4.4	0.45						
			SD	0.10	245	5073	5260	2200	104	1052	125	1933	0.7	0.8	0.3	0.14						
**McM4**	**P**	**1**	**Mean**	0.37	1254	26573	25319	17746	3156	4980	3168	8797	5.2	7.8	6.8	0.31						
n=59			***SD***	0.12	584	15639	15218	12721	1722	3463	1728	8965	0.7	1.0	0.6	0.11						
	**P**	**2**	**Mean**	1.30	1348	22927	21579	4809	4836	3945	3246	4965	4.3	7.3	4.4	0.82						
			SD	0.12	335	16081	15979	2418	2529	1450	216	2186	0.9	0.9	0.7	0.07						
	**Bi**	**3**	**Mean**	1.10	5199	10607	5408	4098	6836	7250	6414	8250	3.9	5.3	4.5	0.83	5199	8192	1	2	2.1	0
			SD	0.10	1131	770	1226	1203	1893	1553	1985	673	0.9	0.8	0.6	0.18	1131	790	0	0	0.5	0
		**Entire**	**Mean**	1.66	1091	26521	25430	7426	4836	3965	3258	5020	4.8	7.4	5.0	1.08						
			SD	0.16	465	15555	15331	8278	2529	1474	239	2217	1.2	1.0	0.5	0.13						
**McM5**	**W**	**1**	**Mean**	0.41	4963	10589	5626	2079	6215	6171	5890	6596	2.4	6.1	3.2	0.31	10589	5029	0.4	0.4	1.0	0
n=84			***SD***	0.09	644	2274	2149	1781	1261	1299	1143	1793	0.2	0.6	0.5	0.10	2274	674	1	1	2.2	0
	**P**	**2**	**Mean**	0.23	1360	11226	9865	5824	2824	3127	2358	4831	4.5	6.8	5.8	0.18						
			SD	0.13	903	3626	3476	2065	1709	1317	851	1742	0.6	0.4	0.5	0.11						
		**Entire**	**Mean**	0.66	1365	12255	10890	6416	6356	5950	4693	7006	4.3	6.9	4.6	0.47						
			SD	0.14	897	3299	3169	3040	2974	1083	1648	2612	1.3	0.4	0.5	0.13						
**McM5a**	**W**	**1**	**Mean**	0.50	5322	9482	4160	2729	6115	6281	5917	6646	2.3	4.6	3.1	0.41	9482	5397	0	0	0.2	0
n=43			***SD***	0.10	1419	1898	1199	1202	1455	1471	1446	1546	0.9	0.9	0.9	0.09	1898	1629	0	0	0.6	1
**McM6**	**P**	**1**	**Mean**	0.10	915	8405	7491	3328	2313	2453	2250	3578	4.0	6.2	5.2	0.10						
n=13			***SD***	0.03	361	998	1323	1219	72	135	81	1001	0.7	0.2	0.6	0.00						
	**P**	**2**	**Mean**	0.69	956	8877	7921	2922	2688	2578	2016	3094	3.4	6.3	4.3	0.47						
			SD	0.18	395	916	814	665	636	406	447	615	0.5	0.2	0.3	0.15						

Call Type	Component Type	Component Number	Statistic	Dur [s]	Fmin [Hz]	Fmax [Hz]	Fdelta [Hz]	BW 90% [Hz]	Fpeak [Hz]	Fcentre [Hz]	Q1F [Hz]	Q3F [Hz]	MinEnt [bits]	MaxEnt [bits]	AvgEnt [bits]	Dur90% [s]	Fstart [Hz]	Fend [Hz]	Ext	Infl	FM	Steps
		**Entire**	**Mean**	0.80	746	8974	8228	2969	2688	2578	2031	3156	3.8	6.3	4.4	0.50						
			SD	0.18	67	748	716	728	636	406	420	638	0.7	0.2	0.4	0.20						
**McM7**	**W**	**1**	**Mean**	1.38	1309	4714	3406	1990	3188	3094	2734	3542	2.0	4.6	2.9	1.15	1309	4650	7	7	5.4	4
n=88			***SD***	0.54	765	1324	1235	876	991	803	658	932	0.5	0.5	0.6	0.49	765	1331	9	10	6.5	4
**McM8**	**P**	**1**	**Mean**	0.81	1511	15779	14268	6181	4540	5826	3917	7232	3.6	6.2	4.6	0.66						
n=36			***SD***	0.08	733	5399	5479	984	1340	930	578	1138	0.4	0.7	0.4	0.05						
	**Bi**	**2**	**Mean**	0.83	4386	11219	6833	4078	5136	6690	5538	7527	2.8	5.7	3.8	0.64	5016	7204	12	13	15.9	0
			SD	0.09	223	915	1027	725	529	1231	674	1092	0.3	0.5	0.3	0.05	719	983	2	2	2.9	0
		**Entire**	**Mean**	0.84	1408	15930	14522	6194	4821	5792	3897	7199	3.5	6.5	4.6	0.69						
			SD	0.08	722	5255	5412	972	1030	824	556	1126	0.4	0.5	0.4	0.07						
**McM9**	**P**	**1**	**Mean**	0.63	4368	7098	2730	1113	6773	6797	6656	6902	2.6	5.1	2.6	0.50	4461	5501	3	4	5.6	2
n=19			***SD***	0.21	620	654	333	864	814	721	730	709	0.7	0.4	0.2	0.14	635	339	1	2	2.0	1
	**Bi**	**2**	**Mean**	0.36	915	18218	17303	6773	3457	3562	2941	5016	3.3	7.4	4.7	0.28						
			SD	0.06	445	835	522	3970	234	115	449	2178	0.3	0.5	0.3	0.05						
		**Entire**	**Mean**	0.99	873	18364	17490	6129	5379	5508	4195	6832	3.4	7.4	3.9	0.78						
			SD	0.25	322	852	570	2729	2404	2028	2094	772	0.7	0.4	0.5	0.21						
**McM10**	**P**	**1**	**Mean**	0.07	1905	19587	17681	4084	4932	4805	4321	5401	3.3	7.0	4.9	0.05						
n=95			***SD***	0.02	1081	5445	5655	2212	911	828	842	883	0.6	0.9	0.7	0.05						
	**W**	**2**	**Mean**	0.25	4402	7544	3142	2258	5526	5724	5281	6304	2.7	5.4	3.5	0.16	4578	5770	2	2	10.1	0
			SD	0.08	685	1230	993	867	924	879	741	1022	0.5	0.7	0.7	0.06	705	1247	2	2	6.7	0
	**P**	**3**	**Mean**	0.28	955	24704	23749	9025	3027	3586	2406	5303	3.7	7.5	5.4	0.21						
			SD	0.07	522	12452	12721	10074	1787	1557	570	3047	1.2	0.9	0.9	0.05						
		**Entire**	**Mean**	0.54	843	25262	24420	8663	4465	4705	3585	6570	3.4	7.6	5.0	0.41						
			SD	0.11	523	12178	12504	9681	1631	1412	1137	2408	1.1	0.9	0.8	0.11						
**McM11**	**P**	**1**	**Mean**	1.65	413	13998	13585	2894	1690	1892	1548	2678	4.1	7.7	5.4	1.23						
n=36			***SD***	0.38	119	3415	3374	2124	263	188	153	514	1.4	1.0	0.8	0.39						
**McM12**	**P**	**1**	**Mean**	0.75	271	5824	5553	2531	1504	1910	1451	2629	4.8	7.2	6.1	0.58						
n=65			***SD***	0.19	99	404	380	357	568	319	97	503	0.6	0.1	0.3	0.15						
**McM13**	**P**	**1**	**Mean**	0.81	1251	47492	46241	28094	4656	4734	3125	8797	5.9	8.4	7.1	0.60						
n=3			***SD***	0.08	409	544	951	10723	2828	2477	866	4902	0.5	0.9	0.4	0.00						
	**W**	**2**	**Mean**	0.44	5521	7494	1973	1578	6859	6594	6141	6984	3.6	5.0	4.4	0.40	6269	6745	0	0	0.0	0
			SD	0.01	118	362	250	379	882	420	47	665	0.4	0.1	0.4	0.00	1415	945	0	0	0.0	0

Call Type	Component Type	Component Number	Statistic	Dur [s]	Fmin [Hz]	Fmax [Hz]	Fdelta [Hz]	BW 90% [Hz]	Fpeak [Hz]	Fcentre [Hz]	Q1F [Hz]	Q3F [Hz]	MinEnt [bits]	MaxEnt [bits]	AvgEnt [bits]	Dur90% [s]	Fstart [Hz]	Fend [Hz]	Ext	Infl	FM	Steps
**McM14**	**P**	**1**	**Mean**	0.30	278	28188	27910	3636	2745	2842	2001	3516	3.3	7.2	5.0	0.20						
n=16			***SD***	0.10	130	7315	7369	1834	955	496	584	326	0.1	0.2	0.4	0.08						
**McM15**	**W**	**1**	**Mean**	0.53	4542	11385	6843	4492	6507	7762	6800	8305	3.1	6.3	4.6	0.45	10905	5022	5	5	9.2	0
n=89			***SD***	0.15	1668	2636	2212	2110	2342	1625	1751	1767	0.5	0.5	0.8	0.15	3335	2258	5	5	9.5	1
	**P**	**2**	**Mean**	0.15	1065	15129	14064	3896	3394	3463	2881	4144	3.3	6.8	4.9	0.11						
			SD	0.04	550	6644	6822	1226	782	641	547	610	0.6	0.7	0.4	0.04						
	**P**	**3**	**Mean**	0.33	1835	14652	12817	2639	3408	3560	3298	4095	2.7	7.2	4.2	0.25						
			SD	0.10	729	7203	7272	1571	722	683	566	830	0.4	0.7	0.6	0.09						
		**Entire**	**Mean**	0.99	960	16666	15706	6480	3215	3596	3080	4754	3.2	7.4	5.0	0.74						
			SD	0.20	484	7170	7276	2998	619	503	494	1306	1.0	0.6	0.5	0.22						
**McM15a**	**P**	**1**	**Mean**	0.16	622	21200	20578	4275	3384	3422	2934	3862	2.6	6.5	4.1	0.12						
n=54			***SD***	0.06	211	6329	6357	2131	308	301	451	476	0.5	0.8	0.5	0.04						
	**P**	**2**	**Mean**	0.35	1879	21000	19121	4144	3234	3183	3000	3956	1.8	6.9	3.1	0.27						
			SD	0.08	532	4795	4644	4465	865	551	444	968	0.5	0.5	0.8	0.07						
		**Entire**	**Mean**	0.51	599	22273	21674	4486	3422	3347	2873	3914	2.8	6.9	3.5	0.42						
			SD	0.09	229	5076	5109	2813	304	359	326	606	1.7	0.5	0.7	0.08						
**McM16**	**P**	**1**	**Mean**	0.42	1193	15750	14557	2652	3134	2826	2451	3502	2.9	6.2	3.7	0.27						
n=7			***SD***	0.07	394	6030	5899	600	1291	855	890	841	1.6	0.4	0.9	0.10						
**McM17**	**P**	**1**	**Mean**	0.65	857	25578	24721	6078	2109	2844	2156	4359	3.6	6.2	4.3	0.50						
n=3			***SD***	0.12	166	1091	1251	3342	366	1382	430	2268	0.8	0.1	0.8	0.10						
	**Bi**	**2**	**Mean**	0.59	5540	13063	7523	3313	6016	6656	6125	7547	2.7	5.8	4.1	0.43	7977	8831	7	8	14.0	0
			SD	0.18	3289	1090	3069	985	3382	3851	3484	4357	0.2	0.5	0.5	0.06	6141	2165	3	3	1.1	0
		**Entire**	**Mean**	0.66	880	25615	24735	6078	2109	2844	2156	4359	3.5	6.2	4.3	0.50						
			SD	0.13	186	1078	1255	3342	366	1382	430	2268	0.8	0.1	0.8	0.10						
**McM18**	**P**	**1**	**Mean**	0.26	1821	29203	27382	9125	14359	16047	13969	18313	3.6	7.5	6.4	0.20						
n=3			***SD***	0.10	790	16317	15705	6100	19677	19374	19252	20831	0.5	1.5	1.7	0.10						
	**P**	**2**	**Mean**	1.22	1305	11467	10161	6875	4469	5125	2969	6313	3.8	5.9	4.6	1.00						
			SD	0.04	222	2361	2162	4441	2907	3357	871	3217	1.2	0.9	1.1	0.10						
	**Bi**	**3**	**Mean**	1.09	6163	12938	6775	5500	8516	9078	8313	10844	3.2	5.7	4.0	0.87	7050	9404	12	12	11.1	0
			SD	0.21	1275	523	1530	1597	683	404	423	1172	0.8	0.8	0.7	0.21	2351	801	3	4	3.6	0
		**Entire**	**Mean**	1.48	1305	29175	27870	16531	14234	14703	12531	17484	4.0	6.9	5.3	1.00						
			SD	0.06	247	16269	16091	18337	19789	19703	17321	20664	1.7	1.4	1.6	0.26						

Call Type	Component Type	Component Number	Statistic	Dur [s]	Fmin [Hz]	Fmax [Hz]	Fdelta [Hz]	BW 90% [Hz]	Fpeak [Hz]	Fcentre [Hz]	Q1F [Hz]	Q3F [Hz]	MinEnt [bits]	MaxEnt [bits]	AvgEnt [bits]	Dur90% [s]	Fstart [Hz]	Fend [Hz]	Ext	Infl	FM	Steps
**McM19**	**P**	**1**	**Mean**	0.33	1668	36294	34626	19723	3820	5496	4184	9410	4.5	7.8	6.5	0.28						
n=4			***SD***	0.10	992	7897	8299	12658	4389	3067	2463	3355	1.0	1.0	0.9	0.05						
	**P**	**2**	**Mean**	0.93	1406	9125	7720	6668	4711	4629	3023	6152	2.9	5.4	4.2	0.63						
			SD	0.18	349	2138	1890	1174	3014	2996	1786	3062	1.0	0.5	0.8	0.15						
	**Bi**	**3**	**Mean**	0.78	7907	11747	3840	1523	8883	9141	8883	9633	2.1	4.9	2.5	0.58	9792	8635	3	4	4.5	0
			SD	0.14	1725	2072	2181	1472	835	532	811	673	0.7	1.2	0.9	0.10	3922	753	1	1	1.8	1
		**Entire**	**Mean**	1.24	830	36215	35385	11742	5496	7277	4242	9176	3.3	6.4	4.9	0.85						
			SD	0.23	492	7880	8171	4006	4161	2703	3239	1010	1.4	1.1	0.9	0.06						
**McM20**	**W**	**1**	**Mean**	1.24	3863	6054	2190	1602	4534	4640	4372	5060	2.8	4.7	3.4	0.96	5166	4480	2	2	1.3	0
n=42			***SD***	0.60	2518	3587	1150	1065	2855	2909	2696	3207	0.8	0.9	1.1	0.49	3292	2681	3	3	1.9	0
**McM21**	**W**	**1**	**Mean**	0.19	5456	7773	2317	1120	5830	5960	5829	6206	2.3	4.8	3.0	0.14	7459	6972	1	1	7.3	0
n=52			***SD***	0.08	3241	4166	1436	1056	3211	3261	3206	3398	0.6	1.0	0.8	0.08	4232	4044	1	0	7.5	0
**McM22**	**P**	**1**	**Mean**	0.28	904	32617	31714	3906	2570	2805	2258	3336	3.7	6.9	5.1	0.20						
n=6			***SD***	0.10	411	16560	16402	1722	418	283	323	359	0.8	1.1	0.9	0.09						
	**W**	**2**	**Mean**	0.97	1284	3190	1906	953	2547	2687	2438	2898	1.6	3.8	2.1	0.75	2032	1339	8	9	10.9	0
			SD	0.41	233	447	535	343	378	348	275	361	0.6	0.7	0.6	0.34	464	311	4	4	5.0	0
	**P**	**3**	**Mean**	0.56	1175	11143	9967	3016	2648	2859	2508	3305	2.7	5.8	4.0	0.43						
			SD	0.32	349	5582	5484	1794	418	284	368	523	0.9	0.8	0.9	0.31						
	**Bi**	**4**	**Mean**	1.08	2978	10481	7502	3875	4219	4977	4188	6070	2.4	5.0	3.3	0.80	3067	8088	4	5	5.2	0
			SD	0.34	1407	1175	1859	2408	2303	2490	2262	3205	0.9	1.2	0.9	0.28	1469	1178	3	3	4.2	0
		**Entire**	**Mean**	1.81	890	32566	31675	4172	2625	2789	2516	3141	2.8	6.3	3.7	1.25						
			SD	0.64	412	15192	15066	2640	267	227	121	222	0.8	1.0	1.0	0.57						
**McM23**	**P**	**1**	**Mean**	0.79	1528	11513	9985	7398	4578	5898	3859	7398	3.4	6.3	5.1	0.62						
n=6			***SD***	0.16	321	2403	2485	2130	2278	2659	1946	2466	0.5	0.3	0.5	0.13						
	**Bi**	**2**	**Mean**	0.79	4562	12581	8019	5930	6680	7047	6477	9062	2.9	5.9	4.8	0.62	4610	10515	7	8	9.6	0
			SD	0.15	1184	497	1030	1007	2018	1811	1711	1717	0.4	0.6	0.4	0.04	1253	2130	2	2	2.2	0
		**Entire**	**Mean**	0.82	1543	13276	11733	8406	4516	6008	3953	7641	3.6	6.4	5.3	0.65						
			SD	0.16	330	1613	1664	2127	2198	2580	2020	2345	0.9	0.3	0.5	0.10						
**McM24**	**P**	**1**	**Mean**	1.04	350	9552	9202	4422	1177	1450	1077	2383	2.9	6.6	4.5	0.77						
n=15			***SD***	0.33	149	2575	2713	3024	436	174	224	717	0.4	0.6	0.5	0.31						
