# Supplementary material for: Cold call: the acoustic repertoire of Ross Sea killer whales (Orcinus orca, Type C) in McMurdo Sound, Antarctica
Source: R Soc Open Sci. 2020 Feb 5;7(2):191228. doi: 10.1098/rsos.191228 (PMC7062068; doi:10.1098/rsos.191228)

Supplementary Material, Appendix 2

Rebecca Wellard[1,2], Robert L. Pitman[3,4], John Durban[5], Christine Erbe[1] (2020).
Cold Call: The Acoustic Repertoire of Ross Sea Killer Whales (*Orcinus orca*, Type C) in McMurdo Sound, Antarctica. *Royal Society Open Science.*

[1] Centre for Marine Science & Technology, Curtin University, GPO Box U1987, Perth, WA 6845, Australia.

[2] Project ORCA, Perth, WA 6026, Australia.

[3] Antarctic Ecosystem Research Division, Southwest Fisheries Science Center, National Marine Fisheries Service, 8901 La Jolla Shores Dr., La Jolla, CA 92037, USA.

[4] Marine Mammal Institute, Oregon State University, 2030 SE Marine Science Drive, Newport, OR 97365, USA.

[5] Marine Mammal and Turtle Division, Southwest Fisheries Science Center, National Marine Fisheries Service, 8901 La Jolla Shores Dr., La Jolla, CA 92037, USA.

This Supplementary Material, Appendix 2 contains the following:

- **Summary.** *Summary on data collection and analysis in this study.*
- **Table 1.** *Information noted during acoustic recordings in McMurdo Sound, Ross Sea between December 2012-2013. Information recorded included GPS location, number of individual killer whales visually counted, behaviours observed, acoustic recording sampling frequency and bit depth, call categories recorded, number of different call categories noted per encounter and the number of individual killer whales identified during each encounter.*
- **Figure 1.** *Spectrogram illustrating how calls are segmented into components.*
- **Figure 2.** *Spectrogram illustrating the parameters that were measured for acoustic analysis.*
- **Figure 3.** *Bar graph illustrating the number of calls repeated per call category by Type C killer whales recorded in McMurdo Sound, Ross Sea, Antarctica.*
- **Call type catalogue** *of vocalisations produced by Type C killer whales recorded in McMurdo Sound, Ross Sea, Antarctica.*

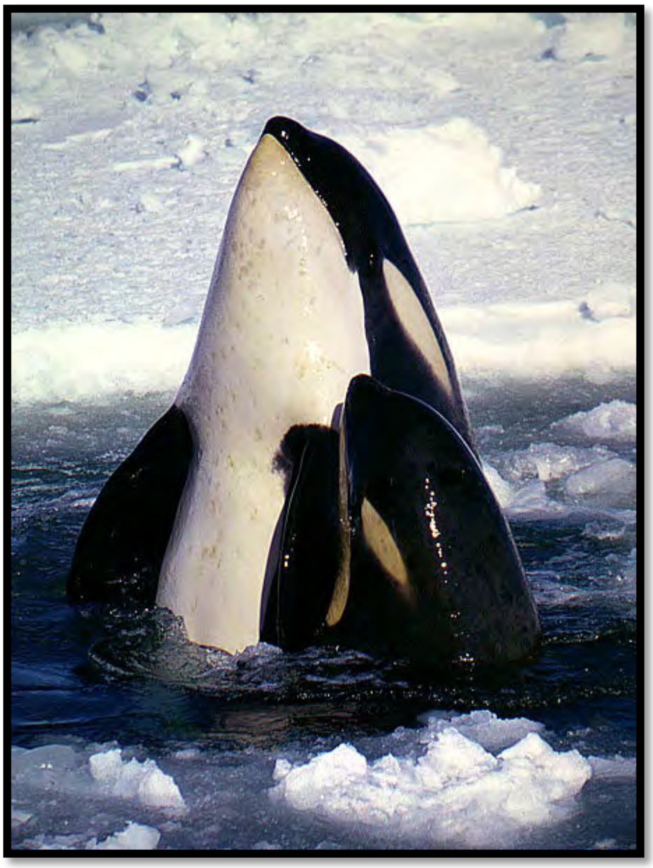

Antarctic Type C Killer Whale Acoustic Call Catalogue from McMurdo Sound, Ross Sea

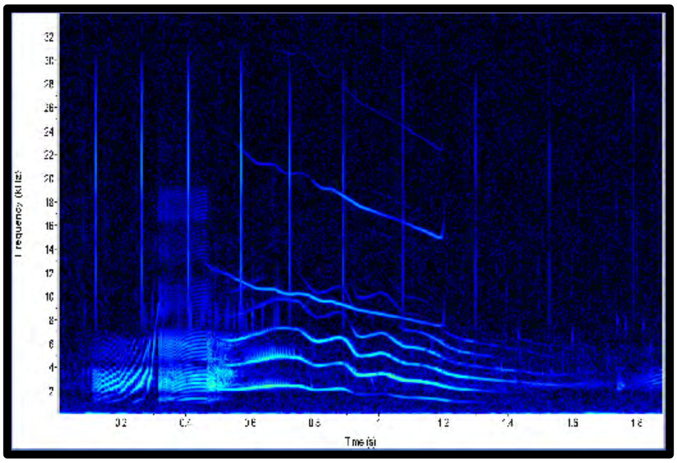

Killer whales (Orcinus orca) are found in all oceans of the world. In Antarctic waters, five ecotypes have been described, each displaying distinct morphological and genetic features, and habitat and diet preferences. Acoustic recordings of Type C killer whales were collected between December 2012 and January 2013 in McMurdo Sound, Ross Sea, Antarctica. Spectrograms of calls were examined for characteristic patterns of Type C vocalisations. Calls were grouped according to their spectro-temporal parameters and a call type catalogue was produced.

Compiled by Rebecca Wellard.

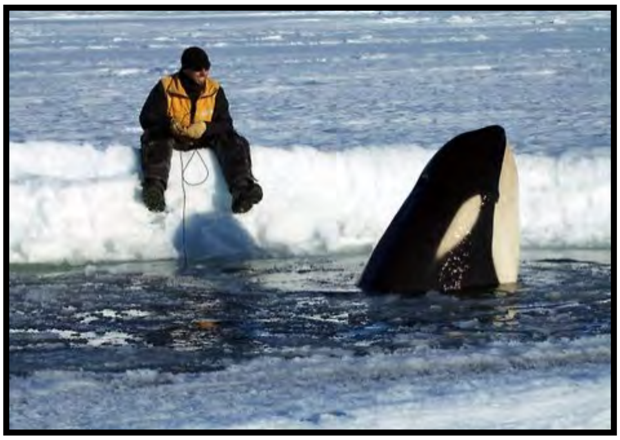

A catalogue of calls produced by Antarctic Type C killer whales (*Orcinus orca*) from McMurdo Sound, Ross Sea

SUMMARY

Acoustic recordings were collected during nine separate encounters with Type C killer whales in McMurdo Sound, Ross Sea. Group size ranged from 8 to 125 individuals, including adults, sub-adults and calves. A total of 392 killer whales were estimated in these sightings, although some of these individuals were likely re-sights while subsurface individuals might have been missed during counting. During each acoustic recording, information on killer whale group composition, number of animals and behavioural was noted (Table 1).

Table 1. Information noted during acoustic recordings in McMurdo Sound, Ross Sea between December 2012-2013. Information recorded included GPS location, number of individual killer whales visually counted, behaviours observed, acoustic recording sampling frequency and bit depth, call categories recorded, number of different call categories noted per encounter and the number of individual killer whales identified during each encounter.

Date	Latitude (°S)	Longitude (°E)	Visual Estimates of Group Sizes	Behaviours observed (F, S, T, M)	Sampling Frequency (kHz)	Bit Depth	Duration of Recordings (mm:ss.0)	Call Categories Recorded	Number of different call categories noted per encounter	Number of Individual Killer Whales Identified
26/12/2012	77.62	165.18	125	F, S	96	24	22:54.4	McM1, McM1a, McM2, McM3, McM3a, McM5, McM5a, McM6, McM7, McM8, McM9, McM10, McM14, McM20, McM21	15	65
29/12/2012	77.62	165.18	19	F, S	96	24	26:44.6	McM3, Mc3a, McM5, McM5a, McM15, McM15a, McM16, McM20	8	19
3/01/2013	77.32	164.56	35	T, S	96	24	06:46.9	McM10	1	31
4/01/2013	77.35	165.5	63	T, S, F, M	96	24	05:34.9	McM1, McM1a, McM2, McM3, McM7, McM8, McM10, McM11, McM14, McM17	10	63
8/01/2013	77.36	165.39	8	T	96	24	15:16.4	McM3, McM4, McM5, McM14, McM20, McM21, McM24	7	7
8/01/2013	77.4	164.95	12	T, F	96	24	22:06.2	McM3, McM3a, McM5, McM10, McM14, McM17, McM20, McM21, McM23	9	6
9/01/2013	77.31	164.54	50	T, F, M	96	24	47:36.3	McM1, McM1a, McM2, McM3, McM3a, McM4, McM5, McM5a, McM7, McM9, McM10, McM13, McM14, McM15, McM15a, McM18, McM19, McM20, McM21, McM22, McM24	21	46
11/01/2013	77.35	165.43	21	T	44.1	24	53:08.0	McM7, McM11, McM12, McM14, McM21, McM24	6	21
11/01/2013	77.36	165.5	59	T, S, F, M	44.1	24	13:03.3	McM7, McM8, McM9, McM10, McM20, McM21,	10	59

A total of 3 hours and 33 minutes of acoustic recordings were analysed from 24 recordings in McMurdo Sound resulting in 6386 killer whale vocalisations detected and subsequently graded. Calls were visually graded based on their signal-to-noise ratio (SNR): Grade 1 ("Poor") if the signal was faint, but still visible; Grade 2 ("Average") if the signal was distinct and clear; and Grade 3 ("Good") if the signal was strong and prominent. Only Grade 2 and 3 calls were selected for analysis and a total of 5134 Grade 1 calls were removed. Categories with fewer than three examples of each type were also removed yielding a total of 1250 vocalisations placed into 28 categories, inclusive of 4 subtypes.

Call categories were principally based on features that are readily discernible in spectrograms, such as the number of successive components (single or multi-component call), duration of the call, presence of simultaneous components (biphonic call) and the overall shape of the call's contour (Figure 1). Calls were classified as biphonic if they had two simultaneous but independently modulated frequency components, otherwise they were classified as monophonic.

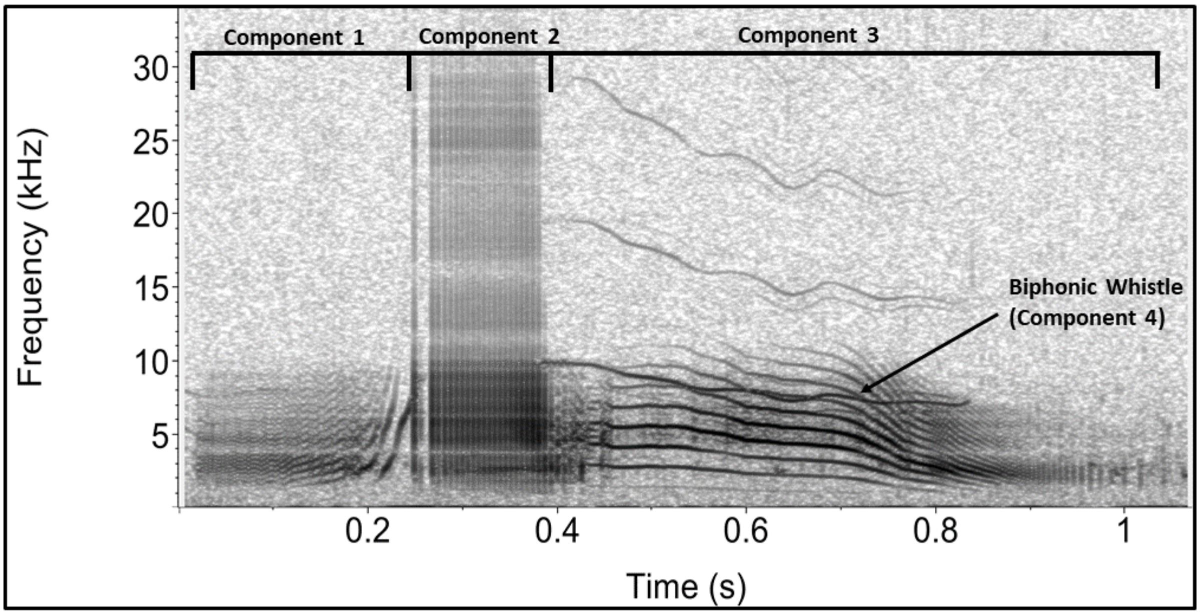

Figure 1. Spectrogram illustrating how calls are segmented into components. This is a 4-component biphonic call categorised as call type McM1. Component 1 is a burst-pulse sound with the pulse repetition rate (PRR) increasing towards the end. The PRR can be read off the spectrogram as the sideband spacing (SBS). Component 2 is a series of single pulses. Components 3 and 4 make up a biphonation. Component 3 is a burst-pulse sound with an SBS of roughly 1 kHz. The PRR decreases towards the end. Component 4 is a whistle with harmonics that decrease in frequency over time (*fs* = *96 kHz, 1024-point FFT, 90% overlap, Hann window*).

Of each call, up to 20 parameters were measured in Raven Pro 1.5 (Bioacoustics Research Program, Cornell University, Ithaca, NY, USA) to quantify its spectro-temporal structure (Supplementary Material, Appendix 1, Table 1). Some of the parameters are more useful for quantifying broadband calls like burst-pulse sounds (e.g., entropy measures and quartile frequencies), while others are more useful for whistles (e.g., start, end, minimum and maximum frequencies of the fundamental contour).

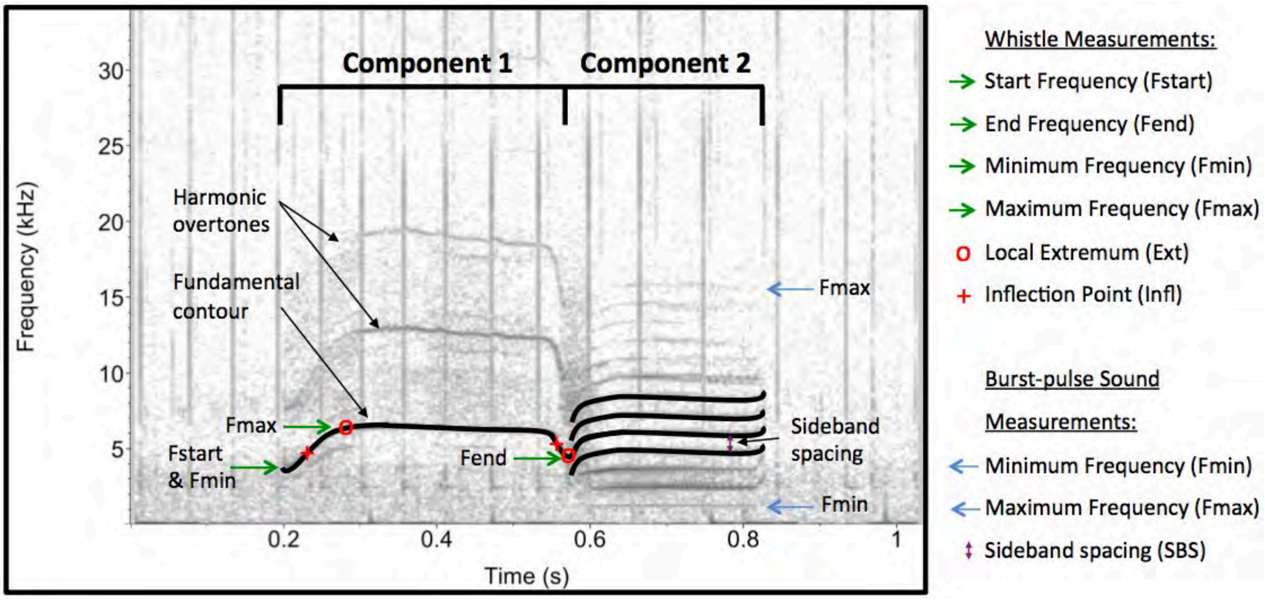

Figure 2. Spectrogram illustrating the parameters that were measured for acoustic analysis. This is a 2-component call categorised as call type McM9. Component 1 is a whistle with harmonics and occasional weak sidebands indicative of amplitude-modulation (AM). Component 2 is a burst-pulse sound with a constant SBS of approximately 1 kHz. Parameters measured off the fundamental contour are illustrated: The start, end, minimum and maximum frequency measurements are labelled by arrows. The local extremum are denoted by a red circle. Inflection points along the contour are denoted by a red cross. The SBS is denoted by a double-ended arrow (*fs = 96 kHz, 1024-point FFT, 90% overlap, Hann window*).

A total of 28 call types are described in this call catalogue, inclusive of four subtypes being variations of the primary call type. The most common call types observed were McM3, McM2, McM1, McM10, McM15, McM7 and McM5 (n=130, 10.4%; n=111, 8.9%; n=101, 8.1%; n=95, 7.6%; n=89, 7.1%; n=88, 7.0%; n=84, 6.7%; respectively), while the other 21 call types comprised the remaining vocalisations analysed (n=552, 44.2%; Figure 3).

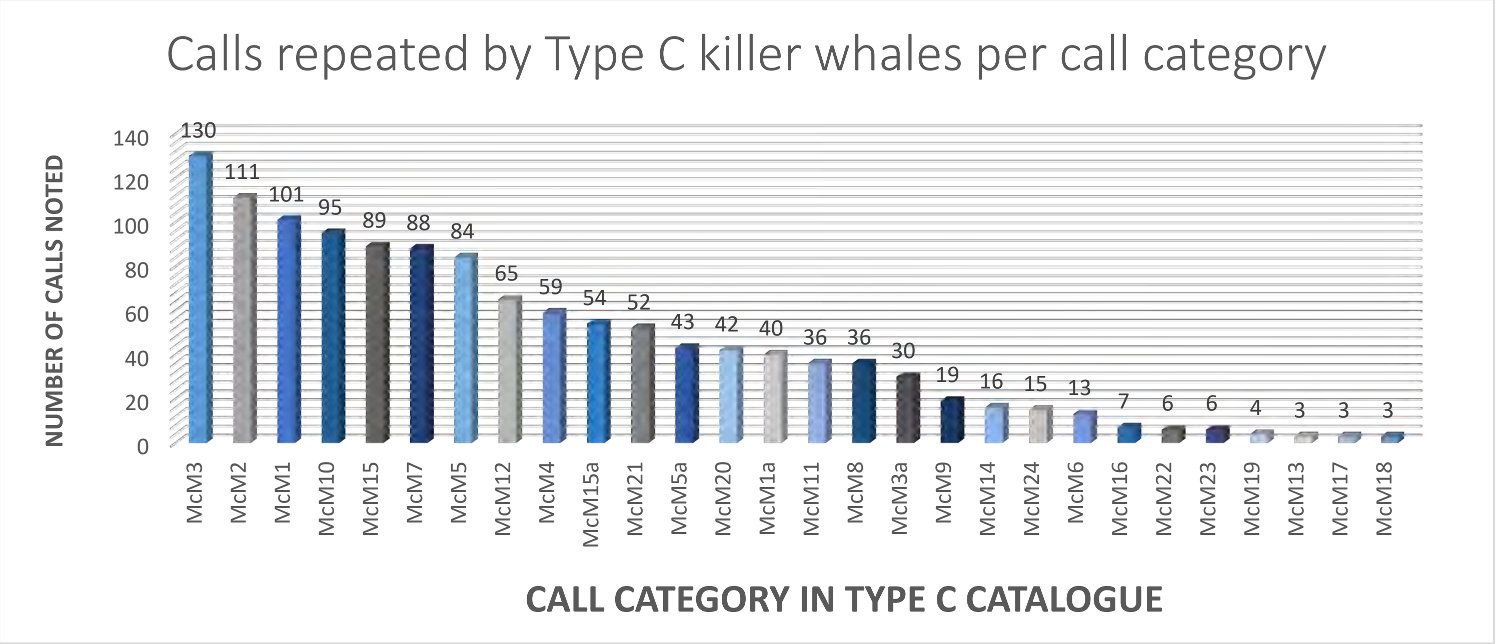

Figure 3. Bar graph illustrating the number of calls repeated per call category by Type C killer whales recorded in McMurdo Sound, Ross Sea, Antarctica.

Four call categories were deemed subtypes. Three of the four subtypes (McM1a, McM5a and McM15a) had a deletion of one or more components from the primary call, whilst the remaining category (3a) had a variation in one of the components' frequency contour.

The number of multi-component calls was higher (n=886, 71%) than the number of single-component calls (n=364, 29%), and 21 out of the 28 call categories consisted of multi-component calls, representative of the complexity of these signals. Of the 28 call categories, 46% were biphonic call categories (n=13) and 54% were monophonic call categories (n=15). In total, there were 532 biphonic calls measured and analysed. This call catalogue provides a spectrogram and a table of acoustic parameters for each call category.

CALL TYPE McM1

This is a 4-component biphonic call. Component 1 is a burst-pulse sound with the PRR increasing towards the end. Component 2 is formed by a series of single pulses. Components 3 and 4 make up a biphonation. Component 3 is a burst-pulse sound with an SBS of roughly 1 kHz. The PRR decreases towards the end and some calls end as a series of distinct pulses. Component 4 is a biphonic whistle with harmonics that decrease in frequency over time. Weak sidebands are sometimes visible indicating AM. Note that the biphonic whistle mostly ends before the biphonic burst-pulse sound does.

n = 101

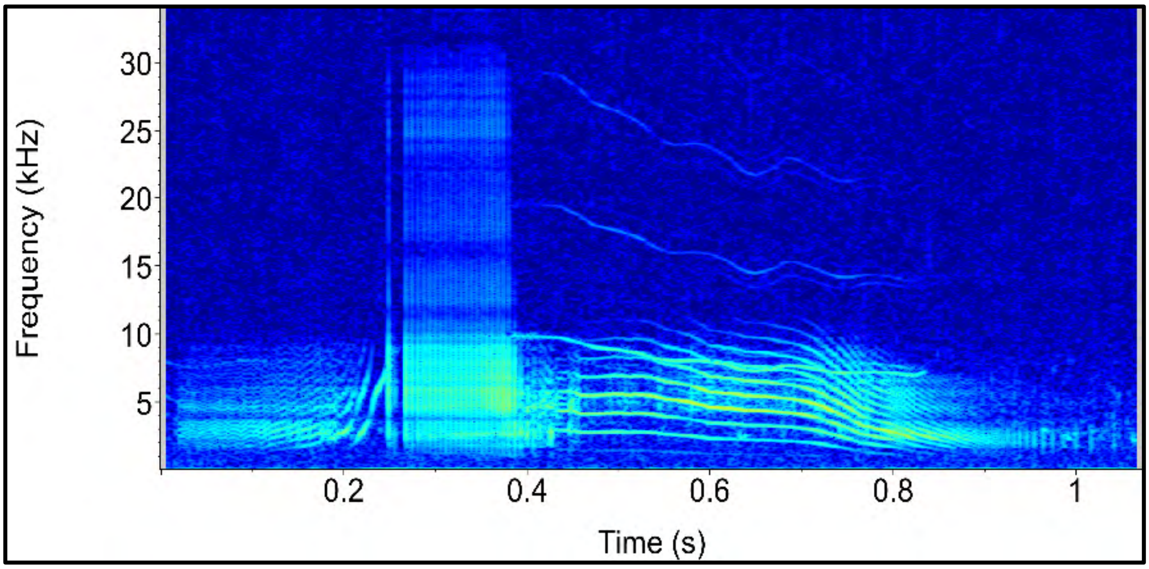

Call Type	Component	Statistic	Dur [s]	Fmin [Hz]	Fmax [Hz]	Fdelta [Hz]	BW 90% [Hz]	Fpeak [Hz]	Fcentre [Hz]	Q1F [Hz]	Q3F [Hz]	MinEnt [bits]	MaxEnt [bits]	AvgEnt [bits]	Dur90% [s]	Fstart [Hz]	Fend [Hz]	Ext	Infl	FM [1/s]	Steps
**McM1**	1	Mean	0.21	1007	12988	11981	3612	2791	3021	2588	3790	3.4	6.8	5.0	0.16						
n=101		*SD*	0.09	478	5032	4997	1790	623	858	396	1301	0.5	0.4	0.5	0.06						
	2	Mean	0.18	830	31350	30521	9743	4073	4402	2951	6354	5.1	7.2	6.2	0.14						
		SD	0.03	367	14593	14686	10008	4041	2185	1190	4682	0.7	0.9	0.8	0.05						
	3	Mean	0.85	884	14401	13517	4502	4155	4071	3012	4852	3.6	6.5	4.3	0.54						
		SD	0.23	321	5315	5407	1840	1140	904	794	880	0.9	0.5	0.4	0.12						
	4	Mean	0.60	7284	12809	5525	3054	8536	8798	8161	9642	2.8	6.0	3.7	0.44	12492	8893	3	3	5.3	0
		SD	0.12	1289	1084	1181	1228	1893	1597	1572	1556	0.7	0.5	0.5	0.10	1826	1746	3	3	5.3	0
	Entire	Mean	1.25	752	30358	29606	5805	4153	4048	2979	4802	3.3	6.9	4.8	0.76						
		SD	0.30	396	13139	13327	3194	1143	952	779	979	0.7	0.8	0.3	0.17						

More examples of Call Type McM1:

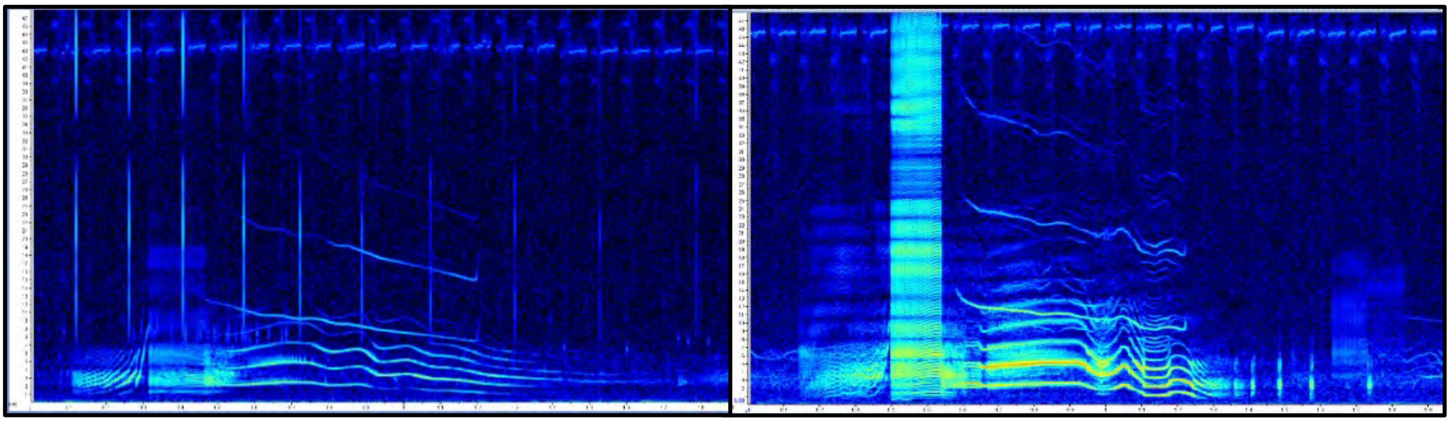

CALL TYPE McM1a

This is a 3-component biphonic call. Due to the first component in Call Type McM1 missing, this call is deemed a variation and hence called Call Type McM1A. Component 1 is formed by a series of single pulses. Component 2 is a burst-pulse sound with a SBS of roughly 1 kHz. The PRR decreases towards the end. Some calls end as series of distinct pulses. Component 3 is a whistle with harmonics and weak sidebands. It decreases in frequency over time. Components 2 and 3 make up the biphonation.

n = 40

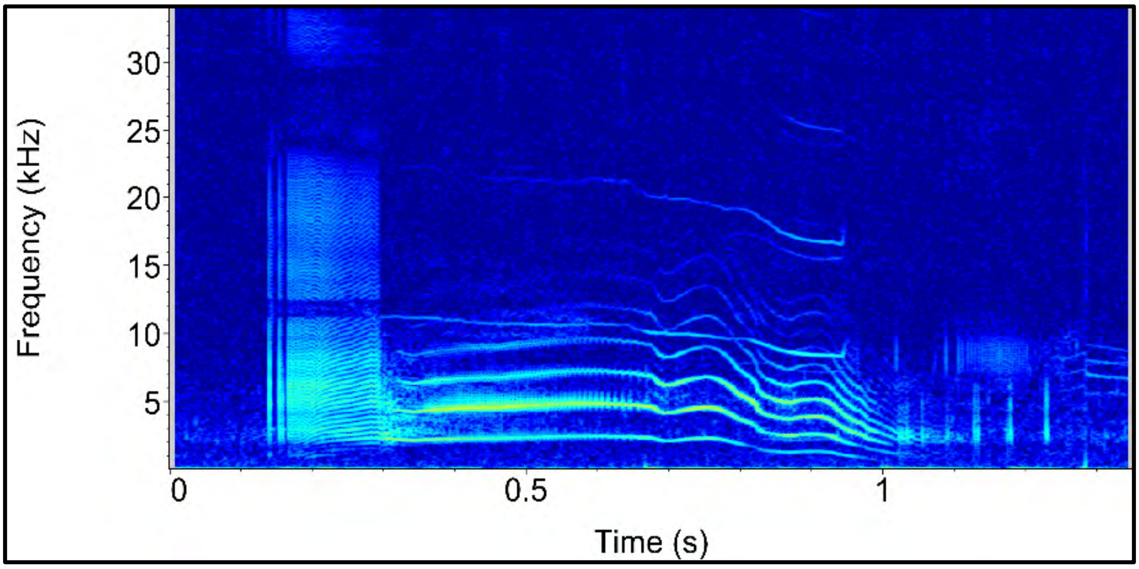

Call Type	Component	Statistic	Dur [s]	Fmin [Hz]	Fmax [Hz]	Fdelta [Hz]	BW 90% [Hz]	Fpeak [Hz]	Fcentre [Hz]	Q1F [Hz]	Q3F [Hz]	MinEnt [bits]	MaxEnt [bits]	AvgEnt [bits]	Dur90% [s]	Fstart [Hz]	Fend [Hz]	Ext	Infl	FM [1/s]	Steps
McM1a	1	Mean	0.22	1634	43989	42355	20361	3188	6352	3609	8824	5.1	8.5	7.2	0.19						
n=40		*SD*	0.04	304	9059	9170	11167	978	4395	1735	4816	1.2	0.6	0.7	0.04						
	2	Mean	0.78	1061	14350	13289	6469	4881	4881	3621	5414	4.1	6.8	4.6	0.60						
		SD	0.14	477	1768	2002	1756	1719	1317	569	1474	0.8	0.4	0.4	0.12						
	3	Mean	0.68	7950	13116	5166	2953	9211	9398	8818	10184	2.8	6.0	3.7	0.54	13116	9390	4	4	5.3	0
		SD	0.12	799	1013	934	630	1271	1045	973	898	0.8	0.4	0.3	0.17	1013	1110	3	2	3.5	0
	Entire	Mean	0.94	907	43937	43030	14133	4881	4922	3650	6768	3.7	7.2	5.3	0.73						
		SD	0.12	615	8997	9387	9608	1719	1318	711	3702	0.8	0.9	0.6	0.10						

More examples of Call Type McM1a:

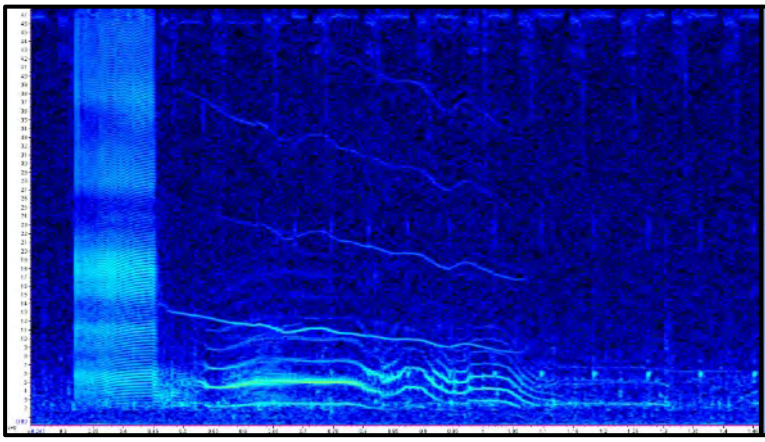

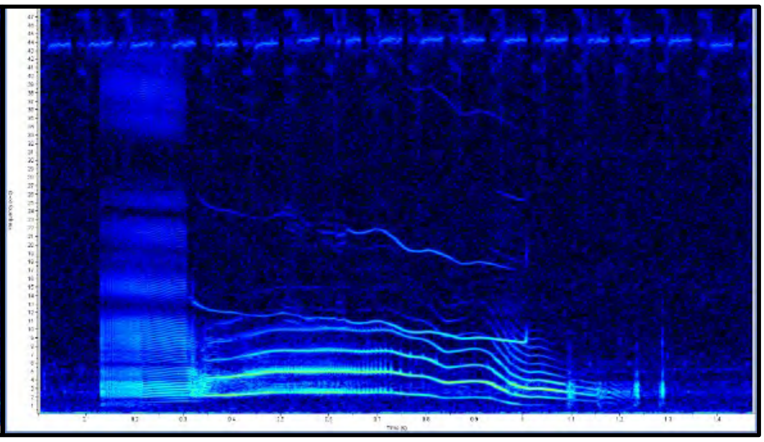

CALL TYPE McM2

This is a 3-component biphonic call. Component 1 is a series of distinct pulses. Component 2 is a burst-pulse sound with an approximate 1 kHz SBS. Component 3 is a whistle with harmonics that first increases in frequency and then decreases in frequency over time. Components 2 and 3 make up a biphonation, although the biphonic whistle ends before the biphonic burst-pulse sound does.

n = 111

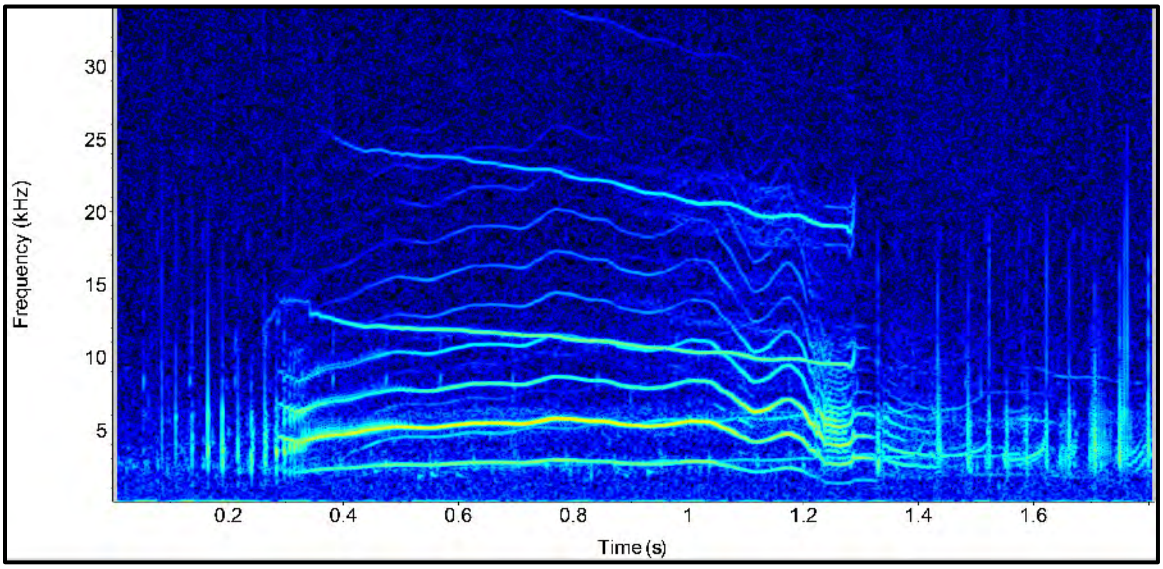

Call Type	Component	Statistic	Dur [s]	Fmin [Hz]	Fmax [Hz]	Fdelta [Hz]	BW 90% [Hz]	Fpeak [Hz]	Fcentre [Hz]	Q1F [Hz]	Q3F [Hz]	MinEnt [bits]	MaxEnt [bits]	AvgEnt [bits]	Dur90% [s]	Fstart [Hz]	Fend [Hz]	Ext	Infl	FM [1/s]	Steps
McM2	1	Mean	0.13	1162	14684	13522	3788	3089	3110	2510	3895	4.0	6.7	5.4	0.10						
n=111		*SD*	0.09	472	8727	8833	1826	1098	865	636	1005	0.8	0.9	0.5	0.06						
	2	Mean	0.82	915	16564	15648	5836	3750	3787	3199	4695	3.5	6.7	4.3	0.58						
		SD	0.17	330	8864	8921	2048	1197	1049	953	931	0.8	0.7	0.4	0.12						
	3	Mean	0.70	7778	12064	4285	2688	9504	9581	8817	10273	2.5	5.3	3.3	0.51	10227	9207	3	4	5.6	0
		SD	0.14	1226	1545	1195	830	1693	1406	1357	1357	0.6	0.5	0.6	0.13	2579	1591	2	3	4.2	0
	Entire	Mean	0.88	986	16115	14924	5878	3766	3769	3183	4685	3.2	6.0	4.2	0.61						
		SD	0.18	420	8783	8178	2006	1211	1047	954	926	0.7	0.6	0.5	0.13						

<u>More examples of Call Type McM2:</u>

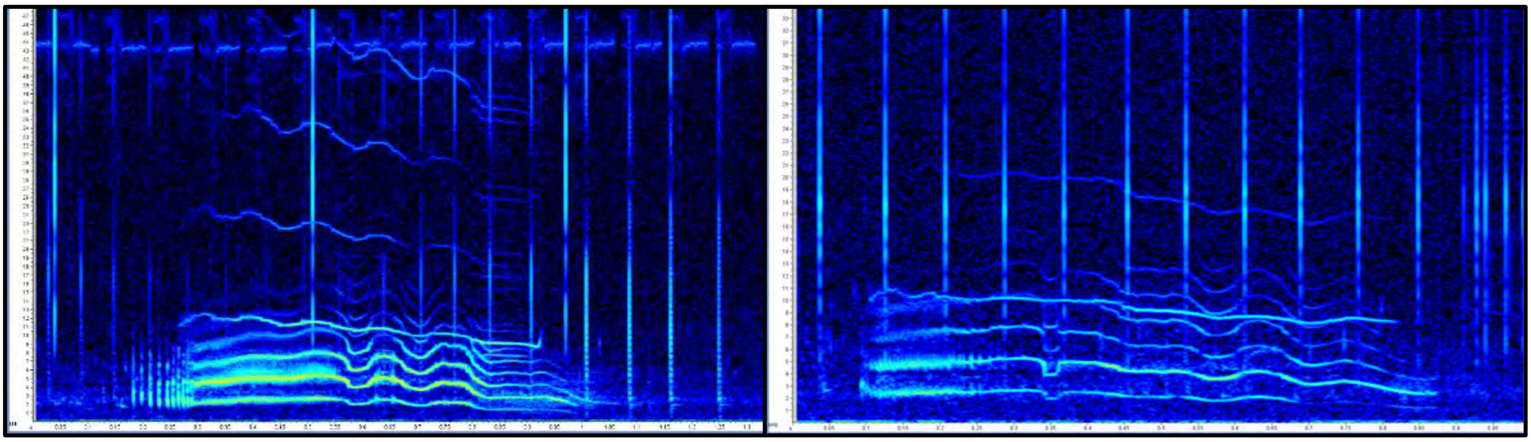

CALL TYPE McM3

This is a 2-component biphonic call. Component 1 is a burst-pulse sound starting at a low SBS (i.e., low PRR), increasing SBS to approximately 1 kHz, and then decreasing SBS towards the end of the component. Component 2 is a simultaneous whistle with harmonics that starts as an upsweep, then remains flat before dropping to a constant wave at a lower frequency.

n = 130

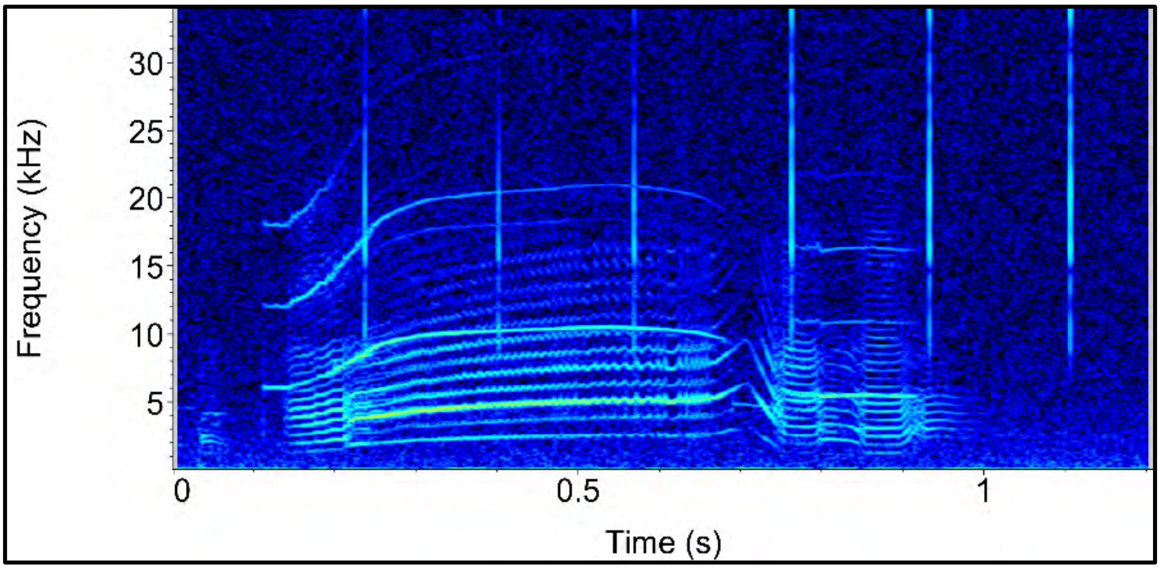

Call Type	Component	Statistic	Dur [s]	Fmin [Hz]	Fmax [Hz]	Fdelta [Hz]	BW 90% [Hz]	Fpeak [Hz]	Fcentre [Hz]	Q1F [Hz]	Q3F [Hz]	MinEnt [bits]	MaxEnt [bits]	AvgEnt [bits]	Dur90% [s]	Fstart [Hz]	Fend [Hz]	Ext	Infl	FM [1/s]	Steps
McM3	1	Mean	0.84	981	17114	16133	7341	3996	4369	3300	6534	3.9	7.2	4.9	0.64						
n=130		*SD*	0.19	376	7939	7963	4150	1857	1913	962	2677	0.9	0.8	0.6	0.14						
	2	Mean	0.61	3628	10666	7037	4686	5661	6120	5248	7511	3.1	5.9	4.1	0.50	4020	7989	2	3	4.8	1
		SD	0.18	1634	1524	1852	2016	2247	2393	1963	2421	0.6	0.7	0.4	0.18	1569	2883	1	1	2.2	1
	Entire	Mean	0.86	987	17328	16341	7425	3984	4373	3308	6533	3.8	7.2	5.0	0.65						
		SD	0.18	388	7674	7723	4044	1867	1911	973	2657	0.9	0.7	0.5	0.14						

More examples of Call Type McM3:

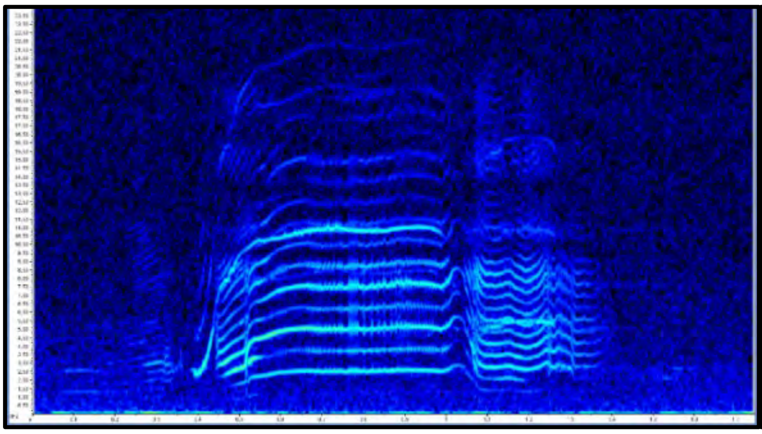

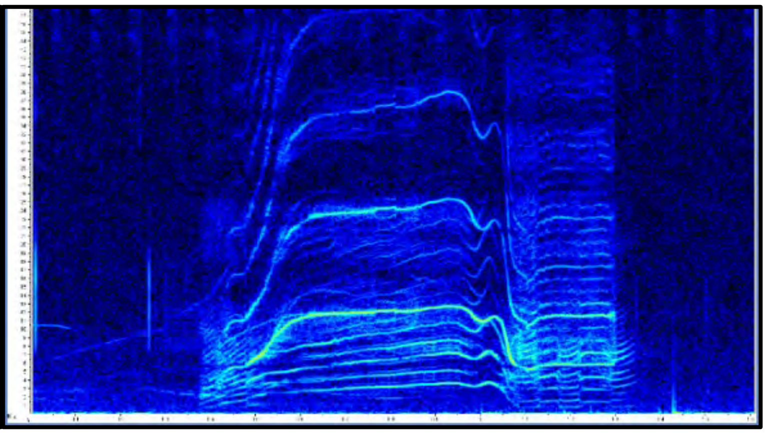

CALL TYPE McM3a

This is a 2-component biphonic call. This call is deemed a variation of Call McM3 due to the first pulse component starting well before the biphonic whistle commences. Component 1 is a burst-pulse sound starting at a low SBS (low PRR), increasing SBS to approximately 1 kHz, then decreasing SBS towards the end of the component. Component 2 is the biphonic whistle with harmonics that consists of an upsweeping contour and then decreases in frequency over time. Note that the biphonic whistle ends before the biphonic burst-pulse sound does.

n = 30

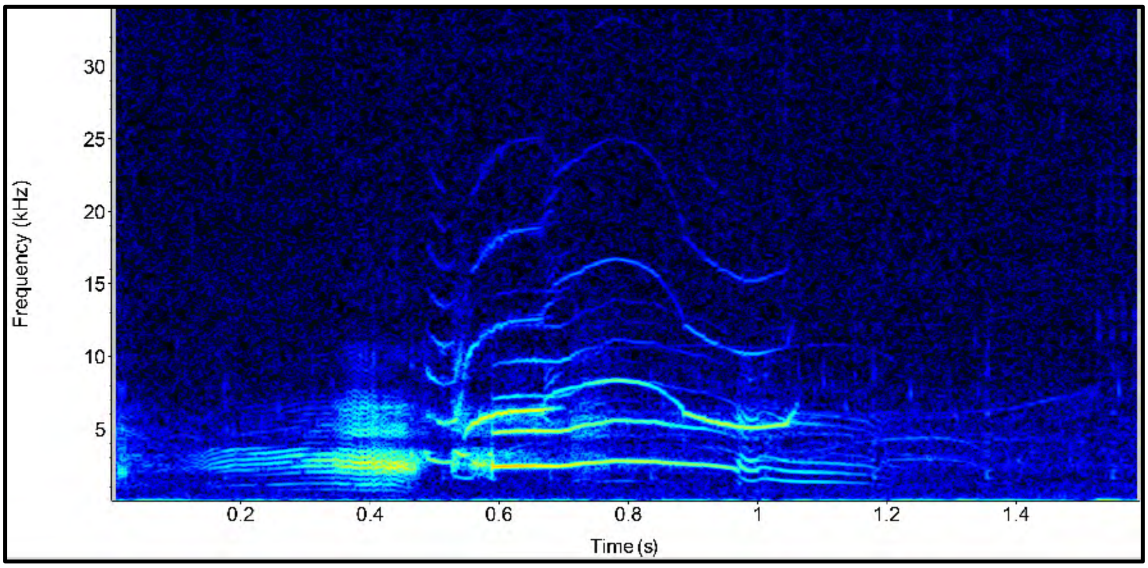

Call Type	Component	Statistic	Dur [s]	Fmin [Hz]	Fmax [Hz]	Fdelta [Hz]	BW 90% [Hz]	Fpeak [Hz]	Fcentre [Hz]	Q1F [Hz]	Q3F [Hz]	MinEnt [bits]	MaxEnt [bits]	AvgEnt [bits]	Dur90% [s]	Fstart [Hz]	Fend [Hz]	Ext	Infl	FM [1/s]	Steps
McM3a	1	Mean	0.98	681	17377	16696	4297	2398	2961	2375	4586	2.9	7.0	4.4	0.45						
n=30		***SD***	0.10	182	5073	5180	2200	104	1052	125	1933	0.7	0.8	0.3	0.14						
	2	Mean	0.45	3178	9832	6654	3742	4250	4898	4102	5656	2.7	6.0	3.6	0.28	3030	7461	2	3	6.8	2
		SD	0.11	1094	1028	1906	2284	1719	1374	1330	1276	0.7	0.3	0.6	0.17	1571	3939	1	1	2.5	1
	Entire	Mean	0.98	612	17316	16704	4297	2398	2961	2375	4586	2.9	7.0	4.4	0.45						
		SD	0.10	245	5073	5260	2200	104	1052	125	1933	0.7	0.8	0.3	0.14						

Another example of Call Type McM3a:

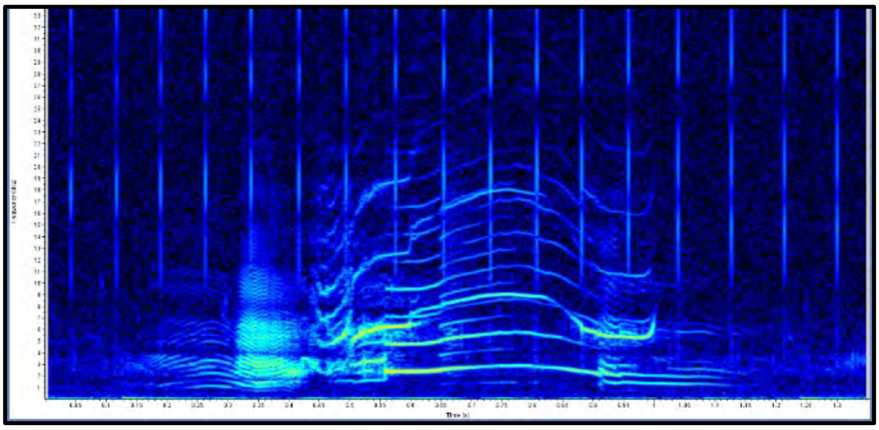

CALL TYPE McM4

This is a 3-component biphonic call. Component 1 consists of a series of distinct pulses. Component 2 is burst-pulse sound with an SBS of up to 2kHz. Component 3 is a whistle with harmonics and weak sidebands (AM) and consists of an upsweeping contour followed by a constant wave. Components 2 and 3 make up the biphonation.

n = 59

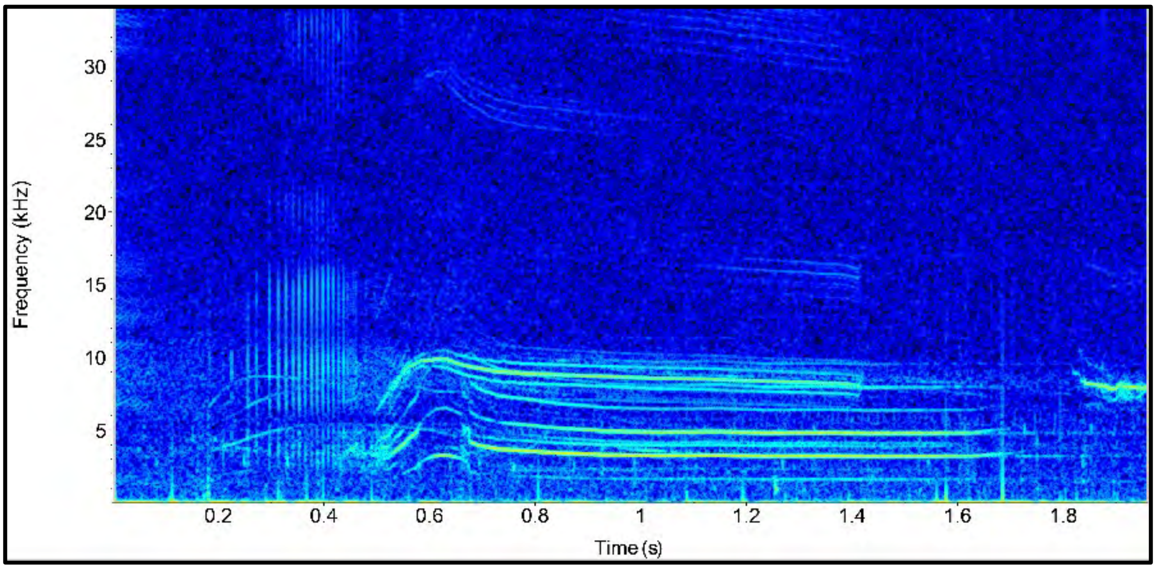

Call Type	Component	Statistic	Dur [s]	Fmin [Hz]	Fmax [Hz]	Fdelta [Hz]	BW 90% [Hz]	Fpeak [Hz]	Fcentre [Hz]	Q1F [Hz]	Q3F [Hz]	MinEnt [bits]	MaxEnt [bits]	AvgEnt [bits]	Dur90% [s]	Fstart [Hz]	Fend [Hz]	Ext	Infl	FM [1/s]	Steps
McM4	1	Mean	0.37	1254	26573	25319	17746	3156	4980	3168	8797	5.2	7.8	6.8	0.31						
n=59		*SD*	0.12	584	15639	15218	12721	1722	3463	1728	8965	0.7	1.0	0.6	0.11						
	2	Mean	1.30	1348	22927	21579	4809	4836	3945	3246	4965	4.3	7.3	4.4	0.82						
		SD	0.12	335	16081	15979	2418	2529	1450	216	2186	0.9	0.9	0.7	0.07						
	3	Mean	1.10	5199	10607	5408	4098	6836	7250	6414	8250	3.9	5.3	4.5	0.83	5199	8192	1	2	2.1	0
		SD	0.10	1131	770	1226	1203	1893	1553	1985	673	0.9	0.8	0.6	0.18	1131	790	0.4	0.4	0.5	0
	Entire	Mean	1.66	1091	26521	25430	7426	4836	3965	3258	5020	4.8	7.4	5.0	1.08						
		SD	0.16	465	15555	15331	8278	2529	1474	239	2217	1.2	1.0	0.5	0.13						

More examples of Call Type McM4:

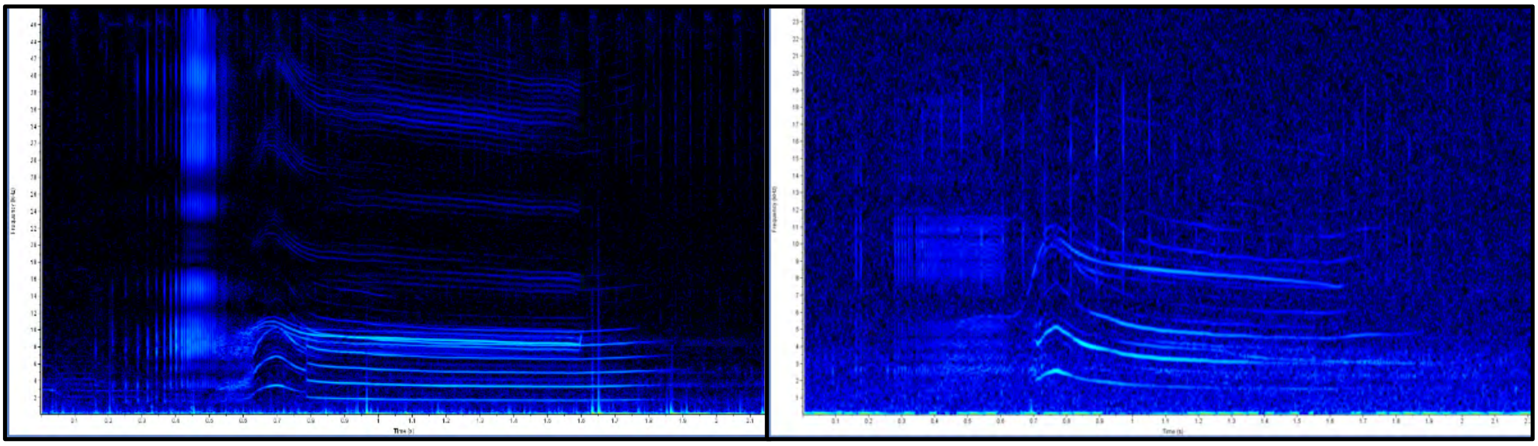

CALL TYPE McM5

This is a 2-component call. Component 1 is a whistle with harmonics starting as a downsweep and ending as a constant wave. Component 2 is burst-pulse sound with SBS (PRR) ranging from approximately 500 Hz to 1 kHz.

n = 84

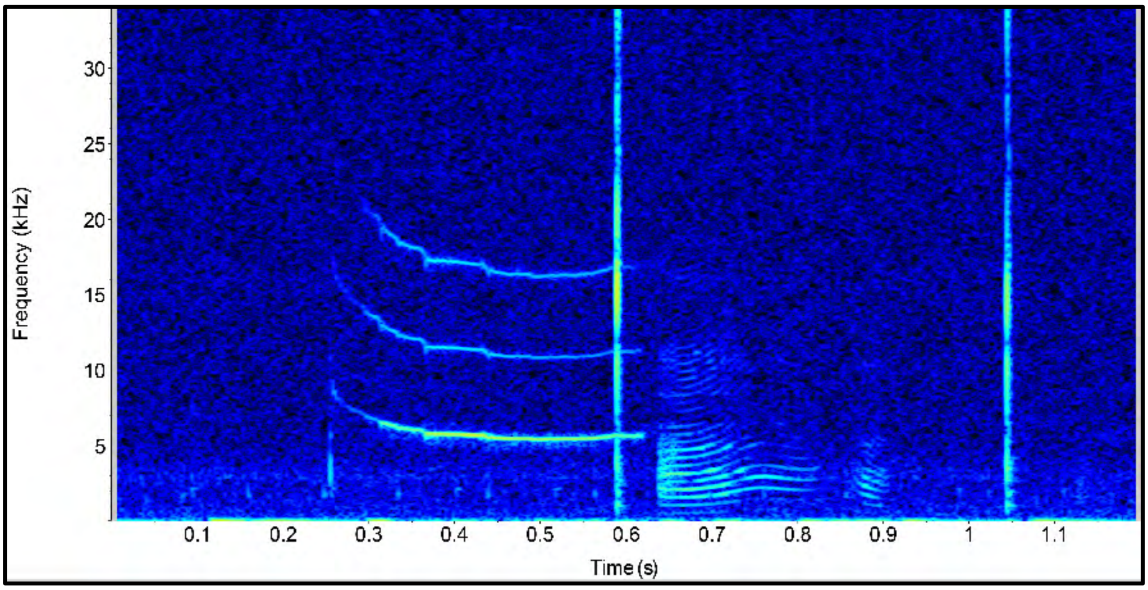

Call Type	Component	Statistic	Dur [s]	Fmin [Hz]	Fmax [Hz]	Fdelta [Hz]	BW 90% [Hz]	Fpeak [Hz]	Fcentre [Hz]	Q1F [Hz]	Q3F [Hz]	MinEnt [bits]	MaxEnt [bits]	AvgEnt [bits]	Dur90% [s]	Fstart [Hz]	Fend [Hz]	Ext	Infl	FM [1/s]	Steps
McM5	1	Mean	0.41	4963	10589	5626	2079	6215	6171	5890	6596	2.4	6.1	3.2	0.31	10589	5029	0.4	0.4	1.0	0
n=84		*SD*	0.09	644	2274	2149	1781	1261	1299	1143	1793	0.2	0.6	0.5	0.10	2274	674	1	1	2.2	0
	2	Mean	0.23	1360	11226	9865	5824	2824	3127	2358	4831	4.5	6.8	5.8	0.18						
		SD	0.13	903	3626	3476	2065	1709	1317	851	1742	0.6	0.4	0.5	0.11						
	Entire	Mean	0.66	1365	12255	10890	6416	6356	5950	4693	7006	4.3	6.9	4.6	0.47						
		SD	0.14	897	3299	3169	3040	2974	1083	1648	2612	1.3	0.4	0.5	0.13						

More examples of Call Type McM5:

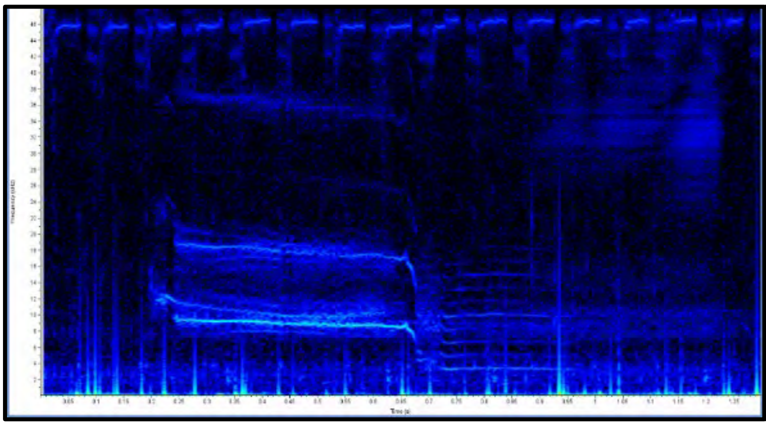

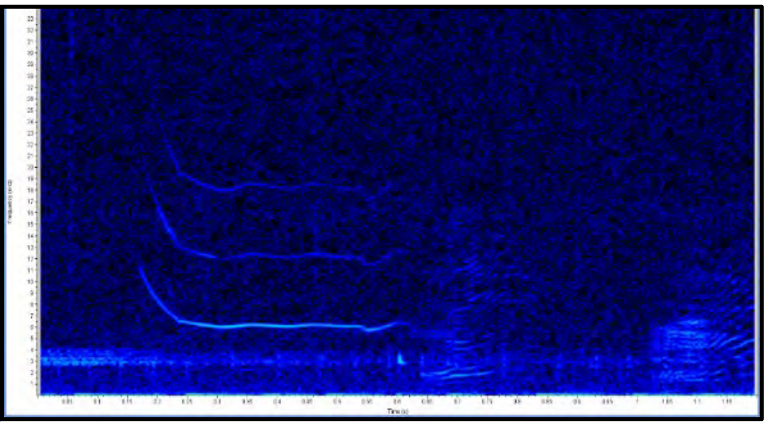

CALL TYPE McM5a

This is a single-component call. This call is deemed a variation of primary call McM5 due to the second component missing. This call consists of a whistle with harmonics starting as a downsweep and ending as a constant wave.

n = 43

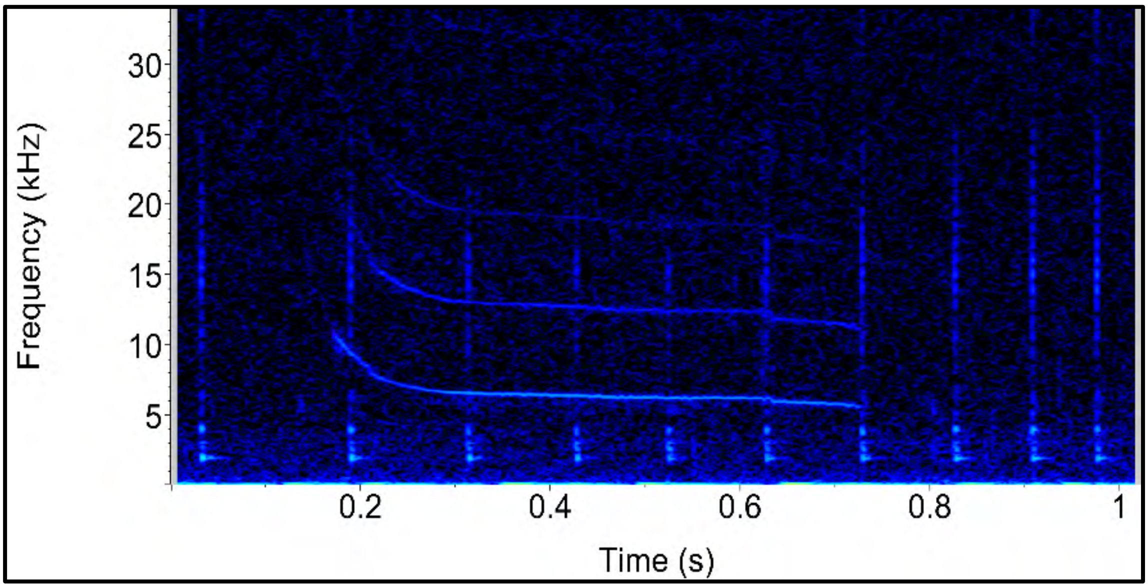

Call Type	Component	Statistic	Dur [s]	Fmin [Hz]	Fmax [Hz]	Fdelta [Hz]	BW 90% [Hz]	Fpeak [Hz]	Fcentre [Hz]	Q1F [Hz]	Q3F [Hz]	MinEnt [bits]	MaxEnt [bits]	AvgEnt [bits]	Dur90% [s]	Fstart [Hz]	Fend [Hz]	Ext	Infl	FM [1/s]	Steps
McM5a	1	Mean	0.50	5322	9482	4160	2729	6115	6281	5917	6646	2.3	4.6	3.1	0.41	9482	5397	0.1	0.1	0.2	0.3
n=43		*SD*	0.10	1419	1898	1199	1202	1455	1471	1446	1546	0.9	0.9	0.9	0.09	1898	1629	0.3	0.3	0.6	1

Another example of Call Type McM5a:

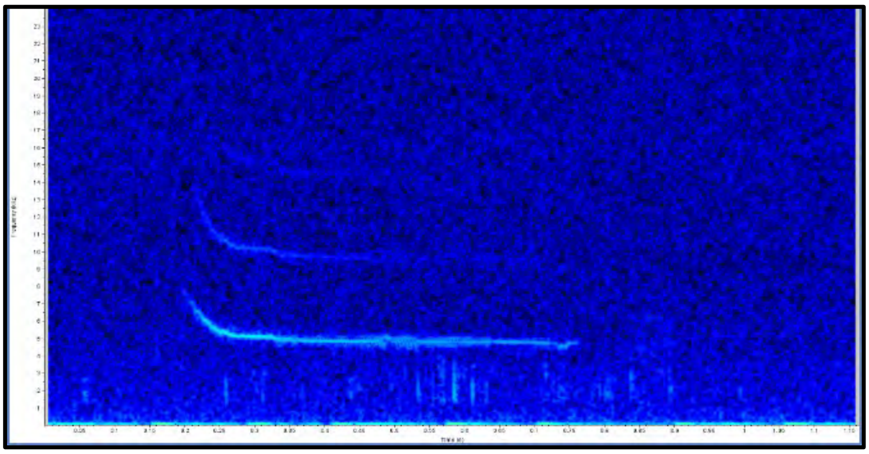

CALL TYPE McM6

This is a 2-component call. Component 1 consists of a series of distinct pulses. As the PRR increases, the spectrogram displays these as an upsweeping contour. Component 2 is burst-pulse sound with a SBS of 1 kHz to 2 kHz, decreasing towards the end.

n = 13

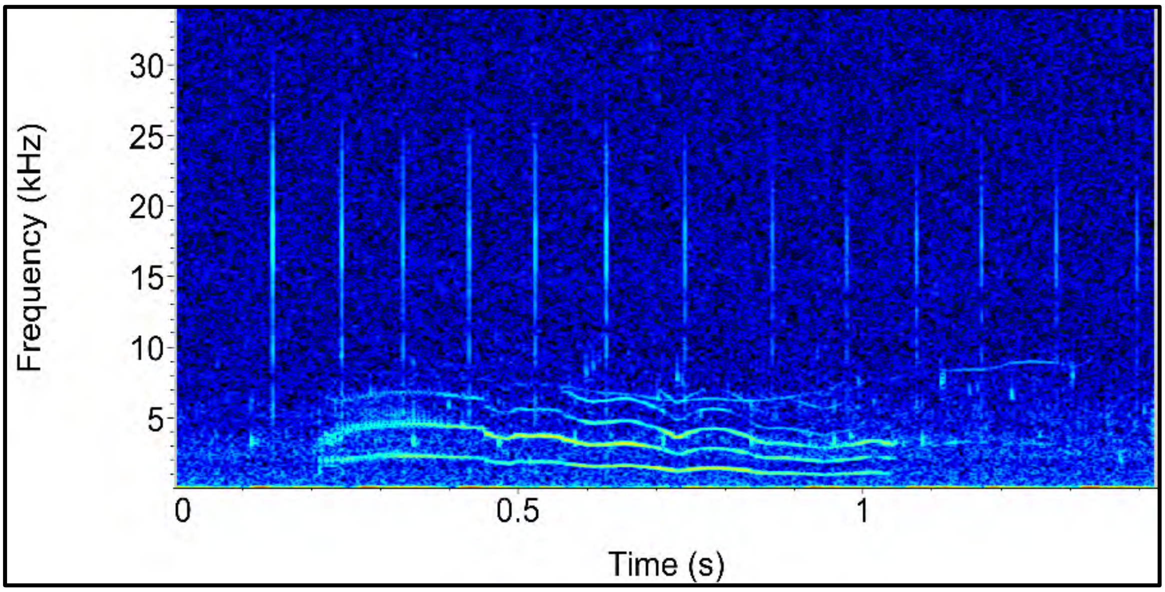

Call Type	Component	Statistic	Dur [s]	Fmin [Hz]	Fmax [Hz]	Fdelta [Hz]	BW 90% [Hz]	Fpeak [Hz]	Fcentre [Hz]	Q1F [Hz]	Q3F [Hz]	MinEnt [bits]	MaxEnt [bits]	AvgEnt [bits]	Dur90% [s]	Fstart [Hz]	Fend [Hz]	Ext	Infl	FM [1/s]	Steps
McM6	1	Mean	0.10	915	8405	7491	3328	2313	2453	2250	3578	4.0	6.2	5.2	0.10						
n=13		*SD*	0.03	361	998	1323	1219	72	135	81	1001	0.7	0.2	0.6	0.00						
	2	Mean	0.69	956	8877	7921	2922	2688	2578	2016	3094	3.4	6.3	4.3	0.47						
		SD	0.18	395	916	814	665	636	406	447	615	0.5	0.2	0.3	0.15						
	Entire	Mean	0.80	746	8974	8228	2969	2688	2578	2031	3156	3.8	6.3	4.4	0.50						
		SD	0.18	67	748	716	728	636	406	420	638	0.7	0.2	0.4	0.20						

Another example of Call Type McM6:

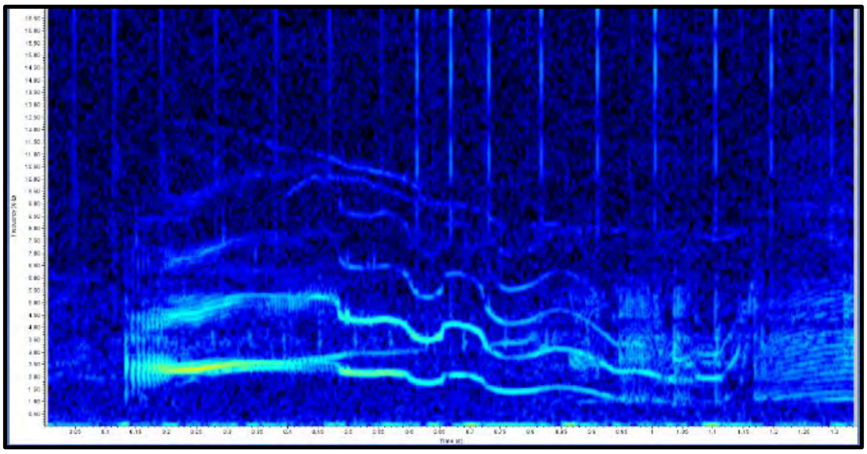

CALL TYPE McM7

This is a singular whistle with a high number of extrema and inflection points and therefore high frequency-modulation. Harmonics are present and some calls start out with distinct pulses and others have steps towards the end of the call. There are signs of AM evidenced by occasional weak sidebands.

n = 88

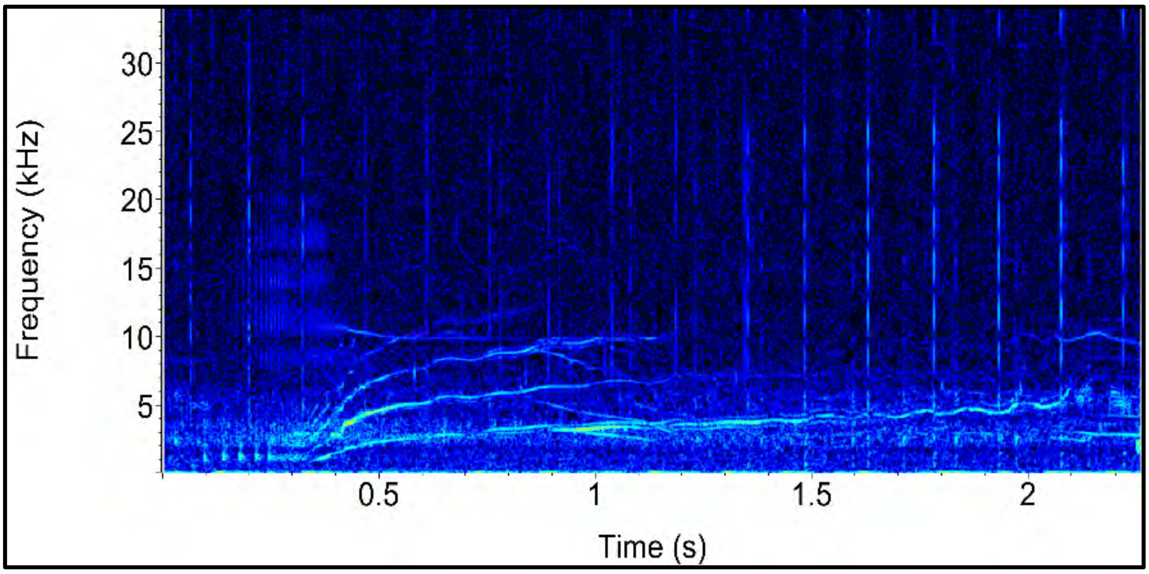

Call Type	Component	Statistic	Dur [s]	Fmin [Hz]	Fmax [Hz]	Fdelta [Hz]	BW 90% [Hz]	Fpeak [Hz]	Fcentre [Hz]	Q1F [Hz]	Q3F [Hz]	MinEnt [bits]	MaxEnt [bits]	AvgEnt [bits]	Dur90% [s]	Fstart [Hz]	Fend [Hz]	Ext	Infl	FM [1/s]	Steps
McM7	1	Mean	1.38	1309	4714	3406	1990	3188	3094	2734	3542	2.0	4.6	2.9	1.15	1309	4650	7	7	5.4	4
n=88		*SD*	0.54	765	1324	1235	876	991	803	658	932	0.5	0.5	0.6	0.49	765	1331	9	10	6.5	4

More examples of Call Type McM7:

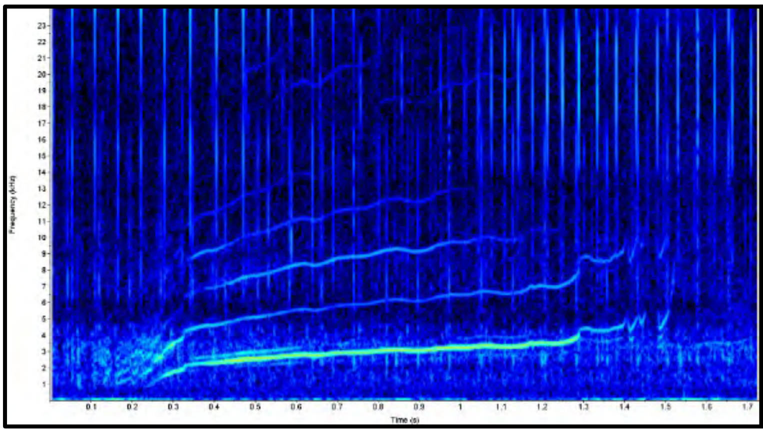

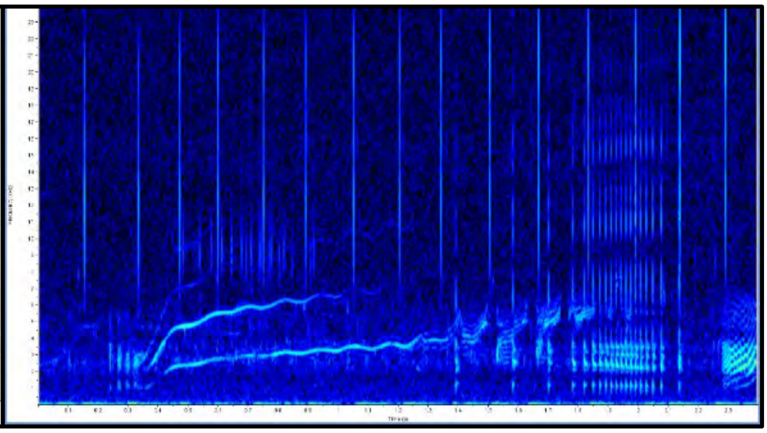

CALL TYPE McM8

This is a 2-component biphonic call. Component 1 is a burst-pulse sound with an SBS of approximately 1 kHz. Component 2 is a simultaneous whistle that is highly frequency-modulated with harmonics that consists of an upsweeping contour followed by a slow downsweep.

n = 36

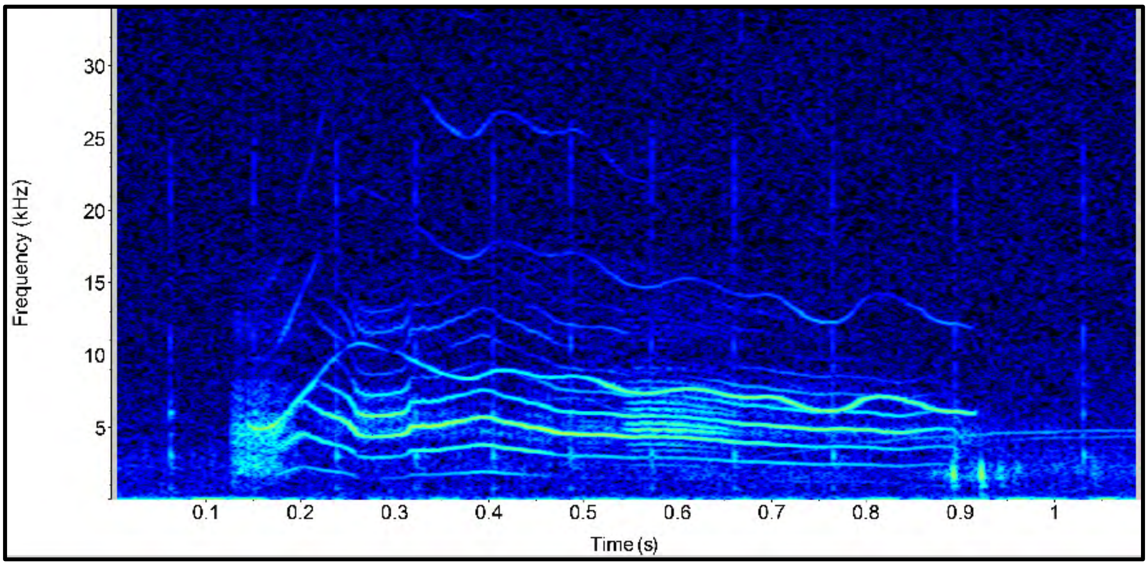

Call Type	Component	Statistic	Dur [s]	Fmin [Hz]	Fmax [Hz]	Fdelta [Hz]	BW 90% [Hz]	Fpeak [Hz]	Fcentre [Hz]	Q1F [Hz]	Q3F [Hz]	MinEnt [bits]	MaxEnt [bits]	AvgEnt [bits]	Dur90% [s]	Fstart [Hz]	Fend [Hz]	Ext	Infl	FM [1/s]	Steps
McM8	1	Mean	0.81	1511	15779	14268	6181	4540	5826	3917	7232	3.6	6.2	4.6	0.66						
n=36		*SD*	0.08	733	5399	5479	984	1340	930	578	1138	0.4	0.7	0.4	0.05						
	2	Mean	0.83	4386	11219	6833	4078	5136	6690	5538	7527	2.8	5.7	3.8	0.64	5016	7204	12	13	15.9	0
		SD	0.09	223	915	1027	725	529	1231	674	1092	0.3	0.5	0.3	0.05	719	983	2	2	2.9	0
	Entire	Mean	0.84	1408	15930	14522	6194	4821	5792	3897	7199	3.5	6.5	4.6	0.69						
		SD	0.08	722	5255	5412	972	1030	824	556	1126	0.4	0.5	0.4	0.07						

<u>More examples of Call Type McM8</u>:

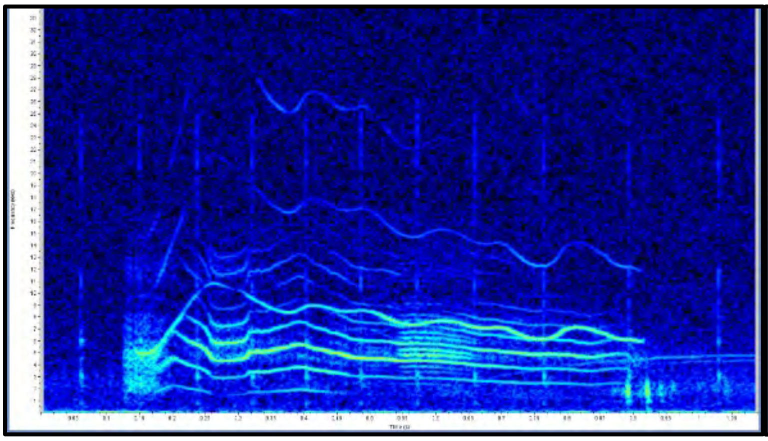

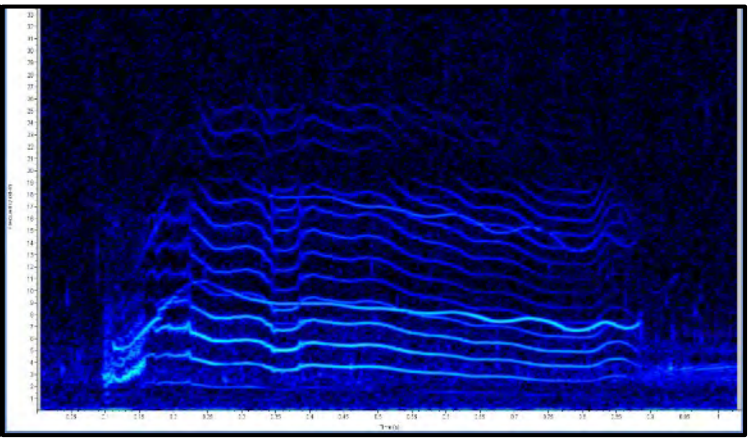

CALL TYPE McM9

This is a 2-component call. Component 1 is a whistle with harmonics and occasional weak sidebands indicative of (AM). Component 2 is a burst-pulse sound with a constant SBS of approximately 1 kHz.

n = 19

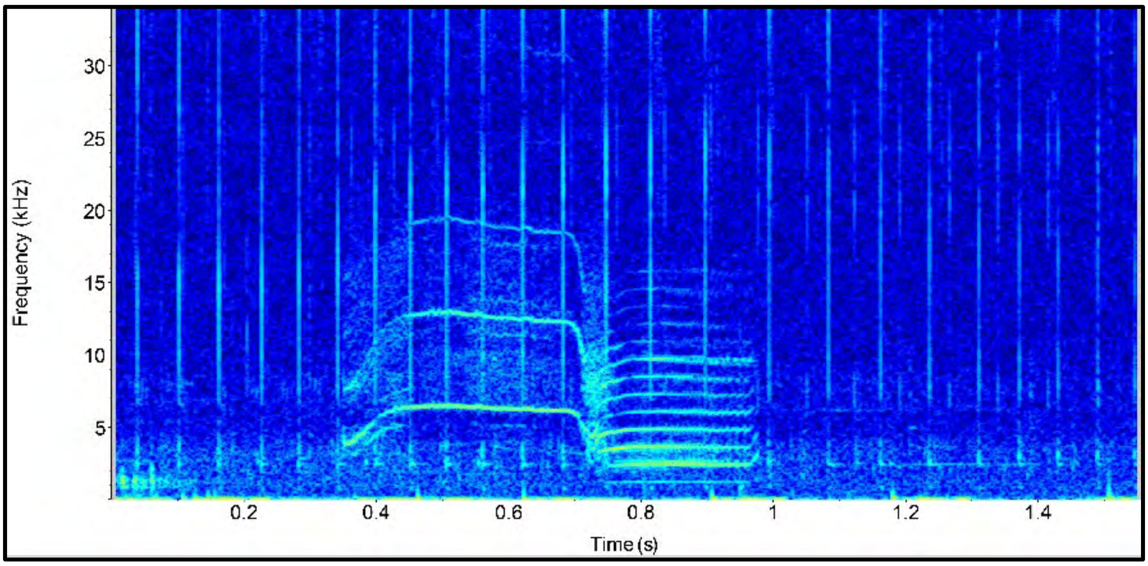

Call Type	Component	Statistic	Dur [s]	Fmin [Hz]	Fmax [Hz]	Fdelta [Hz]	BW 90% [Hz]	Fpeak [Hz]	Fcentre [Hz]	Q1F [Hz]	Q3F [Hz]	MinEnt [bits]	MaxEnt [bits]	AvgEnt [bits]	Dur90% [s]	Fstart [Hz]	Fend [Hz]	Ext	Infl	FM [1/s]	Steps
McM9	1	Mean	0.63	4368	7098	2730	1113	6773	6797	6656	6902	2.6	5.1	2.6	0.50	4461	5501	3	4	5.6	2
n=19		*SD*	0.21	620	654	333	864	814	721	730	709	0.7	0.4	0.2	0.14	635	339	1	2	2.0	1
	2	Mean	0.36	915	18218	17303	6773	3457	3562	2941	5016	3.3	7.4	4.7	0.28						
		SD	0.06	445	835	522	3970	234	115	449	2178	0.3	0.5	0.3	0.05						
	Entire	Mean	0.99	873	18364	17490	6129	5379	5508	4195	6832	3.4	7.4	3.9	0.78						
		SD	0.25	322	852	570	2729	2404	2028	2094	772	0.7	0.4	0.5	0.21						

Another example of Call Type McM9:

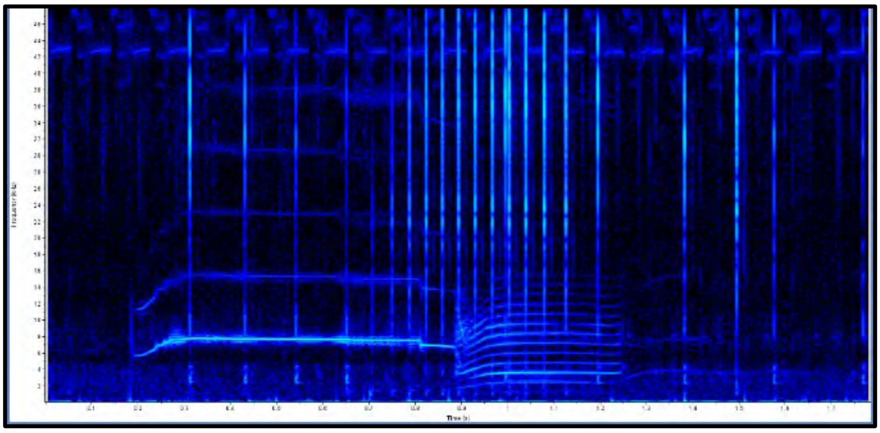

CALL TYPE McM10

This is a 3-component call. Component 1 is a burst-pulse sound. Component 2 is a whistle with harmonics. Component 3 is a burst-pulse sound with a SBS of approximately 1 kHz. Some calls in this category were observed as repeated call sequences, as shown below in the bottom right spectrogram.

n = 95

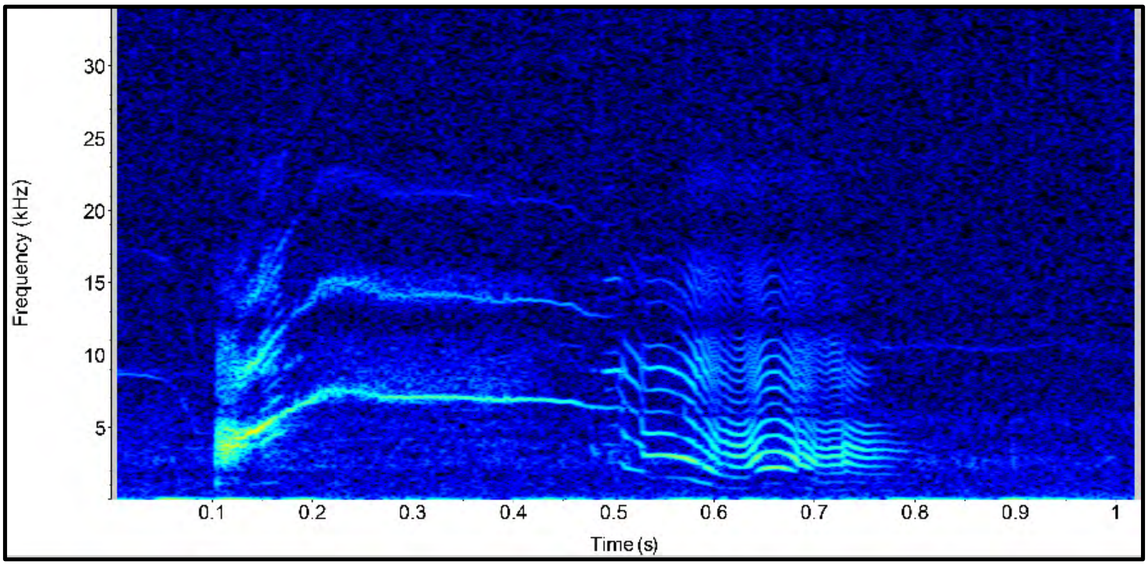

Call Type	Component	Statistic	Dur [s]	Fmin [Hz]	Fmax [Hz]	Fdelta [Hz]	BW 90% [Hz]	Fpeak [Hz]	Fcentre [Hz]	Q1F [Hz]	Q3F [Hz]	MinEnt [bits]	MaxEnt [bits]	AvgEnt [bits]	Dur90% [s]	Fstart [Hz]	Fend [Hz]	Ext	Infl	FM [1/s]	Steps
McM10	1	Mean	0.07	1905	19587	17681	4084	4932	4805	4321	5401	3.3	7.0	4.9	0.05						
n=95		*SD*	0.02	1081	5445	5655	2212	911	828	842	883	0.6	0.9	0.7	0.05						
	2	Mean	0.25	4402	7544	3142	2258	5526	5724	5281	6304	2.7	5.4	3.5	0.16	4578	5770	2	2	10.1	0
		SD	0.08	685	1230	993	867	924	879	741	1022	0.5	0.7	0.7	0.06	705	1247	2	2	6.7	0
	3	Mean	0.28	955	24704	23749	9025	3027	3586	2406	5303	3.7	7.5	5.4	0.21						
		SD	0.07	522	12452	12721	10074	1787	1557	570	3047	1.2	0.9	0.9	0.05						
	Entire	Mean	0.54	843	25262	24420	8663	4465	4705	3585	6570	3.4	7.6	5.0	0.41						
		SD	0.11	523	12178	12504	9681	1631	1412	1137	2408	1.1	0.9	0.8	0.11						

More examples of Call Type McM10:

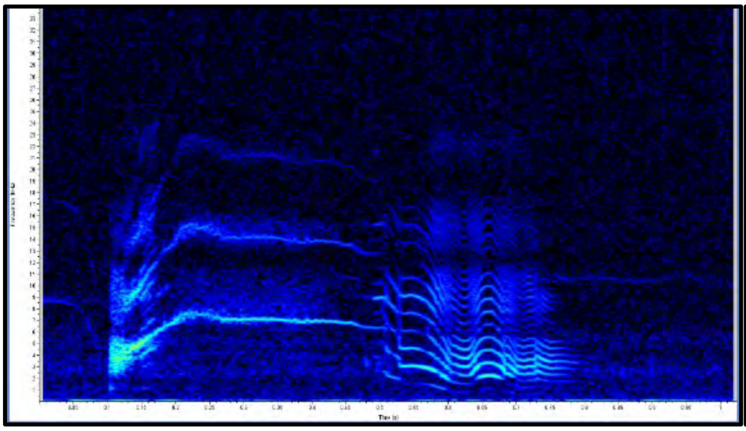

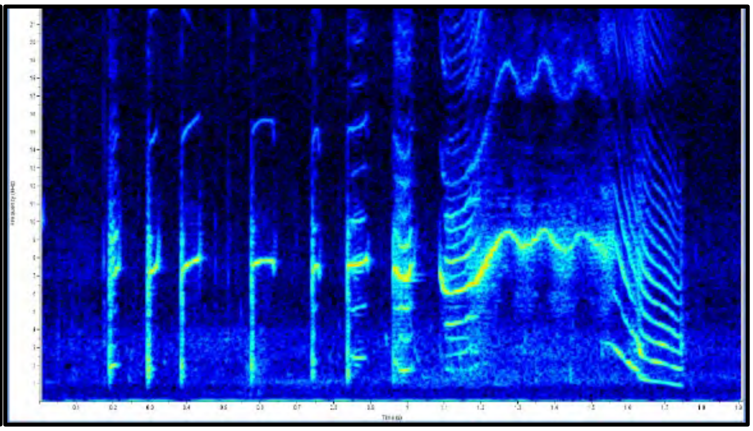

CALL TYPE McM11

This is a single-component call consisting of a burst-pulse sound with a SBS of approximately 500 Hz.
n = 36

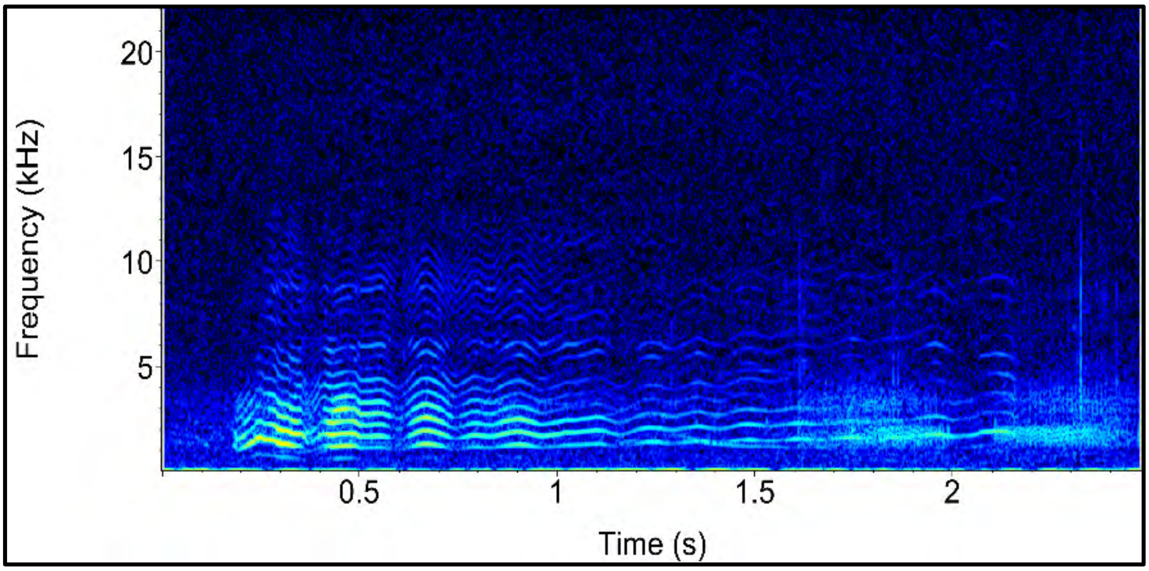

Call Type	Component	Statistic	Dur [s]	Fmin [Hz]	Fmax [Hz]	Fdelta [Hz]	BW 90% [Hz]	Fpeak [Hz]	Fcentre [Hz]	Q1F [Hz]	Q3F [Hz]	MinEnt [bits]	MaxEnt [bits]	AvgEnt [bits]	Dur90% [s]	Fstart [Hz]	Fend [Hz]	Ext	Infl	FM [1/s]	Steps
McM11	1	Mean	1.65	413	13998	13585	2894	1690	1892	1548	2678	4.1	7.7	5.4	1.23						
n=36		***SD***	0.38	119	3415	3374	2124	263	188	153	514	1.4	1.0	0.8	0.39						

<u>Another example of Call Type McM11:</u>

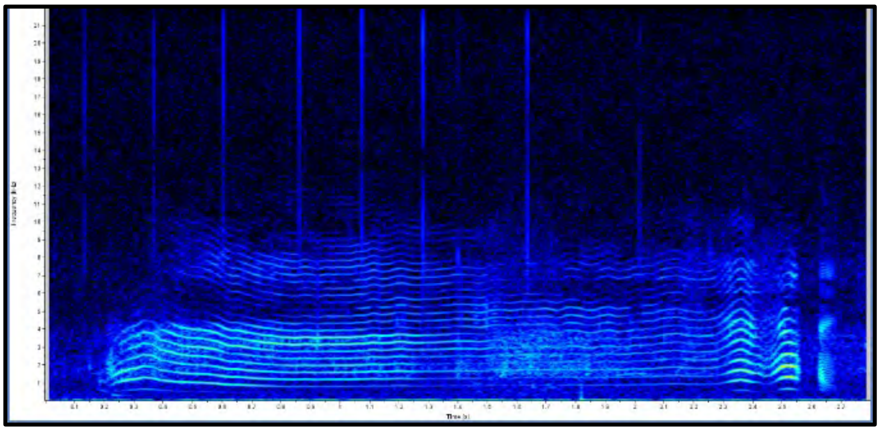

CALL TYPE McM12

**This is a single-component call consisting of a burst-pulse sound starting with an increasing SBS and then decreasing SBS. Calls are often repeated and appear as a ‘reflection’ of each other.**
n = 65

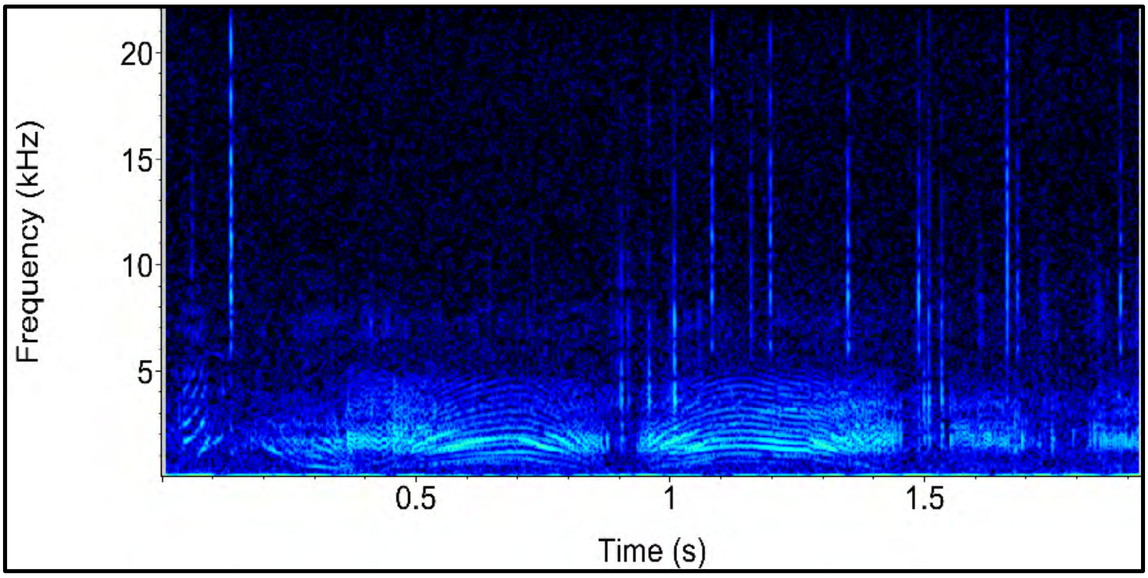

Call Type	Component	Statistic	Dur [s]	Fmin [Hz]	Fmax [Hz]	Fdelta [Hz]	BW 90% [Hz]	Fpeak [Hz]	Fcentre [Hz]	Q1F [Hz]	Q3F [Hz]	MinEnt [bits]	MaxEnt [bits]	AvgEnt [bits]	Dur90% [s]	Fstart [Hz]	Fend [Hz]	Ext	Infl	FM [1/s]	Steps
McM12	1	Mean	0.75	271	5824	5553	2531	1504	1910	1451	2629	4.8	7.2	6.1	0.58						
n=65		***SD***	0.19	99	404	380	357	568	319	97	503	0.6	0.1	0.3	0.15						

Another example of Call Type McM12:

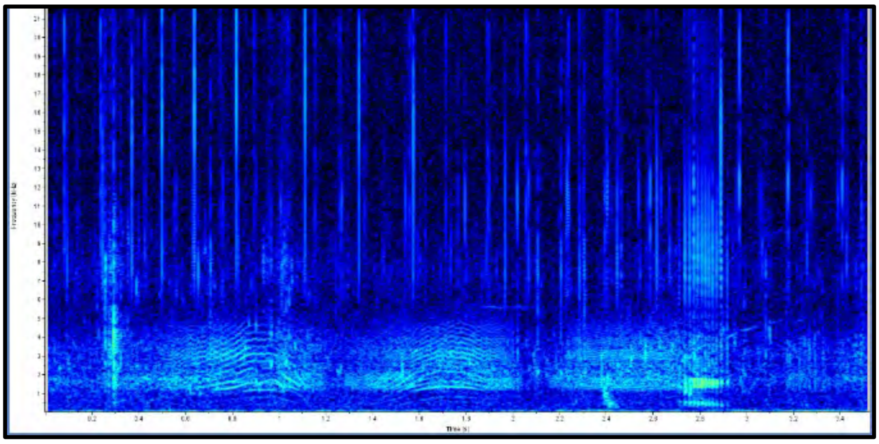

CALL TYPE McM13

This is a 2-component biphonic call. Component 1 starts as distinct pulses at increasing PRR, continuing as a burst-pulse sound. Component 2 is a whistle with harmonics and very little frequency-modulation. Note that the two biphonic components do not have the same duration.

n = 3

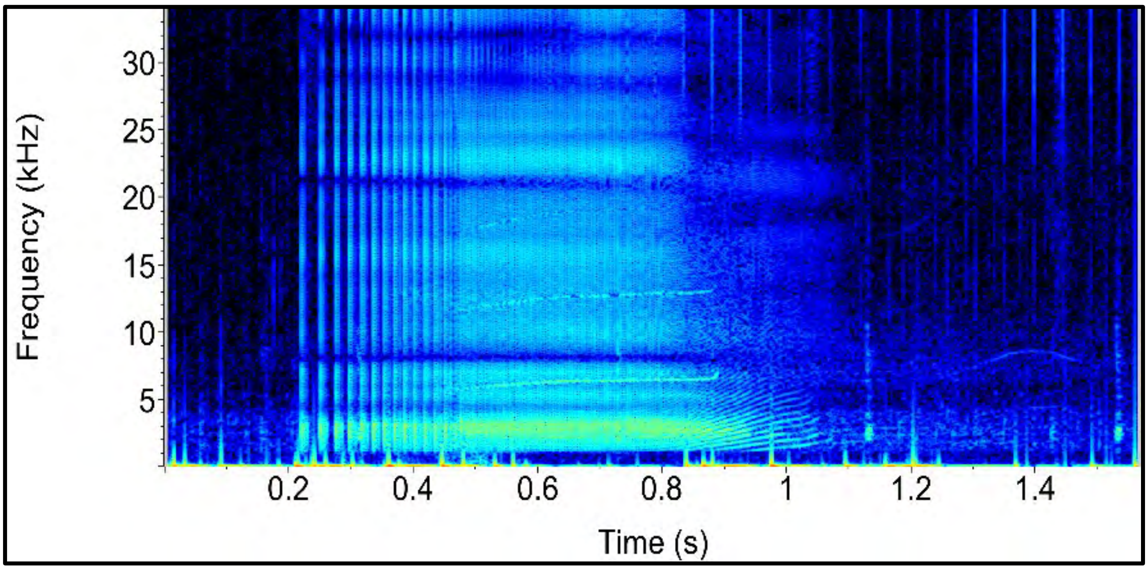

Call Type	Component	Statistic	Dur [s]	Fmin [Hz]	Fmax [Hz]	Fdelta [Hz]	BW 90% [Hz]	Fpeak [Hz]	Fcentre [Hz]	Q1F [Hz]	Q3F [Hz]	MinEnt [bits]	MaxEnt [bits]	AvgEnt [bits]	Dur90% [s]	Fstart [Hz]	Fend [Hz]	Ext	Infl	FM [1/s]	Steps
**McM13**	**1**	**Mean**	0.81	1251	47492	46241	28094	4656	4734	3125	8797	5.9	8.4	7.1	0.60						
n=3		***SD***	0.08	409	544	951	10723	2828	2477	866	4902	0.5	0.9	0.4	0.00						
	**2**	**Mean**	0.44	5521	7494	1973	1578	6859	6594	6141	6984	3.6	5.0	4.4	0.40	6269	6745	0	0	0	0
		SD	0.01	118	362	250	379	882	420	47	665	0.4	0.1	0.4	0.00	1415	945	0	0	0	0

Another example of Call Type McM13:

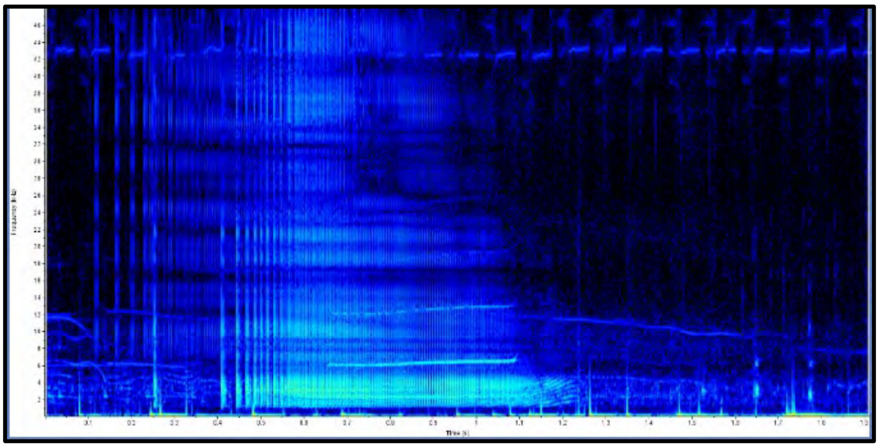

CALL TYPE McM14

This is a single-component burst-pulse sound with increasing SBS (PRR).

n = 16

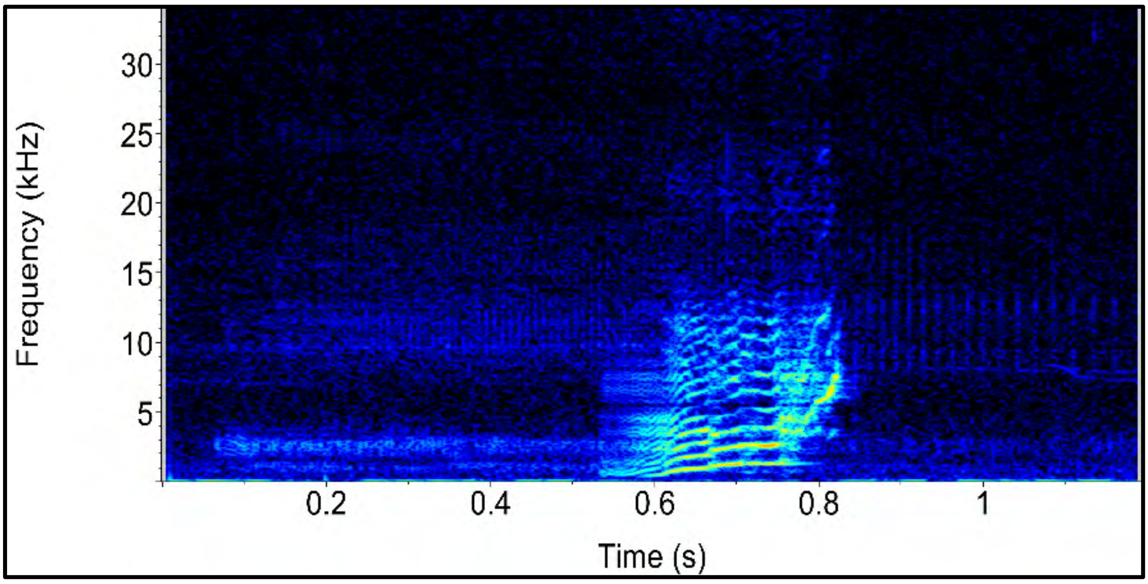

Call Type	Component	Statistic	Dur [s]	Fmin [Hz]	Fmax [Hz]	Fdelta [Hz]	BW 90% [Hz]	Fpeak [Hz]	Fcentre [Hz]	Q1F [Hz]	Q3F [Hz]	MinEnt [bits]	MaxEnt [bits]	AvgEnt [bits]	Dur90% [s]	Fstart [Hz]	Fend [Hz]	Ext	Infl	FM [1/s]	Steps
McM14	1	Mean	0.30	278	28188	27910	3636	2745	2842	2001	3516	3.3	7.2	5.0	0.20						
n=16		*SD*	0.10	130	7315	7369	1834	955	496	584	326	0.1	0.2	0.4	0.08						

Another example of Call Type McM14:

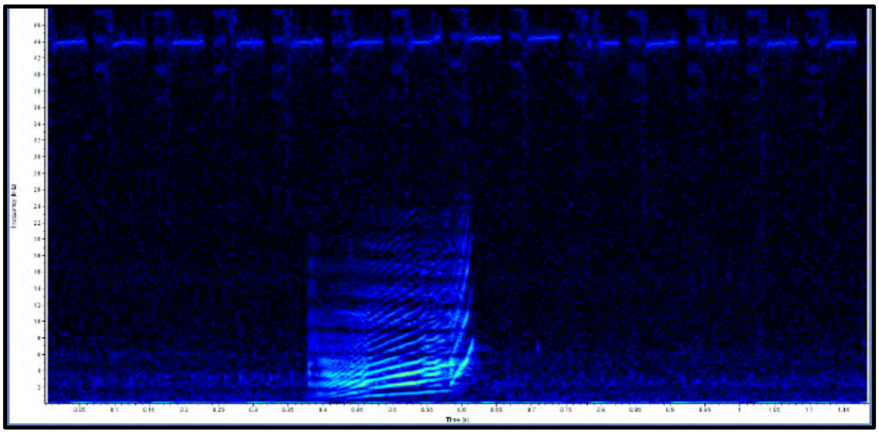

CALL TYPE McM15

This is a 3-component call. Component 1 is a whistle with harmonics. Component 2 a burst-pulse sound with a SBS of 500Hz. Component 3 is another burst-pulse sound with a SBS of 2kHz.

n = 89

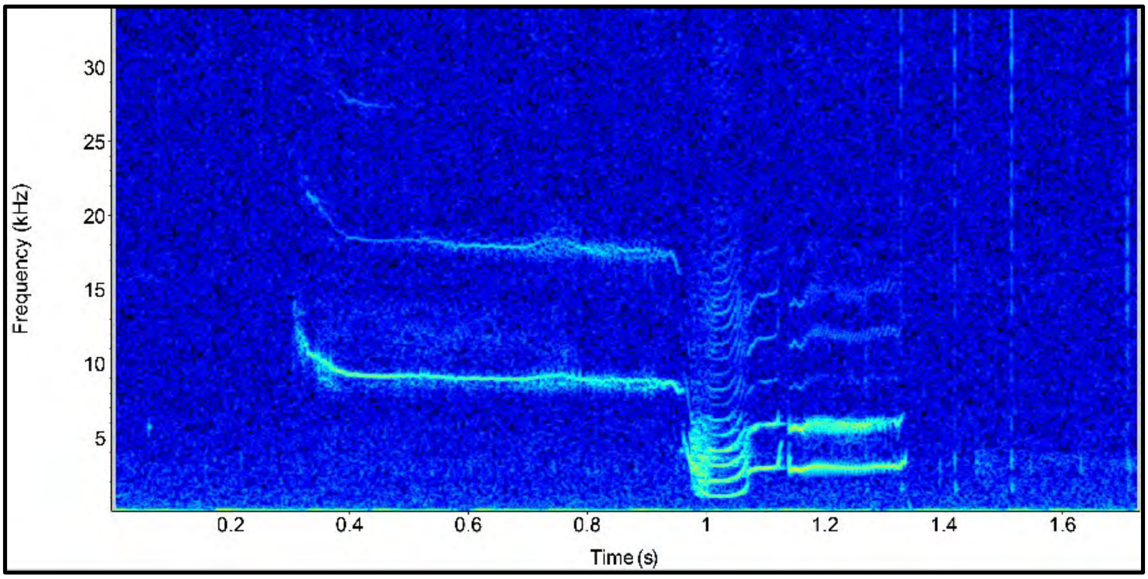

Call Type	Component	Statistic	Dur [s]	Fmin [Hz]	Fmax [Hz]	Fdelta [Hz]	BW 90% [Hz]	Fpeak [Hz]	Fcentre [Hz]	Q1F [Hz]	Q3F [Hz]	MinEnt [bits]	MaxEnt [bits]	AvgEnt [bits]	Dur90% [s]	Fstart [Hz]	Fend [Hz]	Ext	Infl	FM [1/s]	Steps
**McM15**	**1**	**Mean**	0.53	4542	11385	6843	4492	6507	7762	6800	8305	3.1	6.3	4.6	0.45	10905	5022	5	5	9.2	0
n=89		***SD***	0.15	1668	2636	2212	2110	2342	1625	1751	1767	0.5	0.5	0.8	0.15	3335	2258	5	5	9.5	1
	**2**	**Mean**	0.15	1065	15129	14064	3896	3394	3463	2881	4144	3.3	6.8	4.9	0.11						
		SD	0.04	550	6644	6822	1226	782	641	547	610	0.6	0.7	0.4	0.04						
	**3**	**Mean**	0.33	1835	14652	12817	2639	3408	3560	3298	4095	2.7	7.2	4.2	0.25						
		SD	0.10	729	7203	7272	1571	722	683	566	830	0.4	0.7	0.6	0.09						
	**Entire**	**Mean**	0.99	960	16666	15706	6480	3215	3596	3080	4754	3.2	7.4	5.0	0.74						
		SD	0.20	484	7170	7276	2998	619	503	494	1306	1.0	0.6	0.5	0.22						

Another example of Call Type McM15:

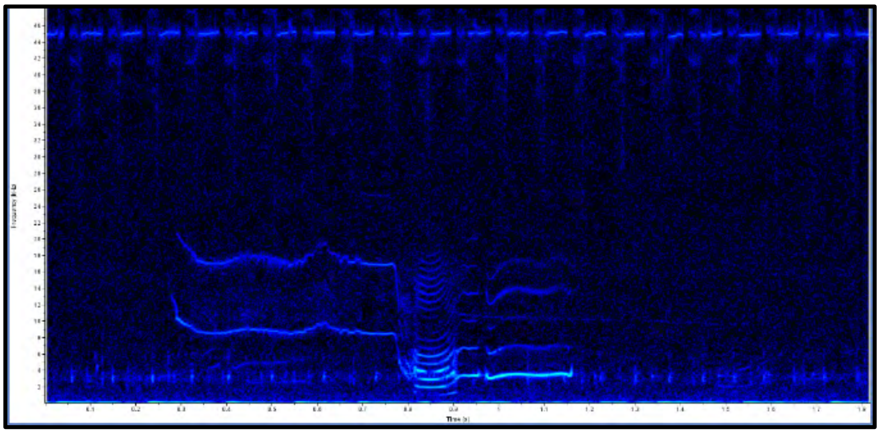

CALL TYPE McM15a

This is a 2-component call and a variation of call type McM15 as the first component of McM15 is missing here. Component 1 is a burst-pulse sound with a SBS of 500Hz. Component 2 is another burst-pulse with a SBS of 2kHz.

n = 54

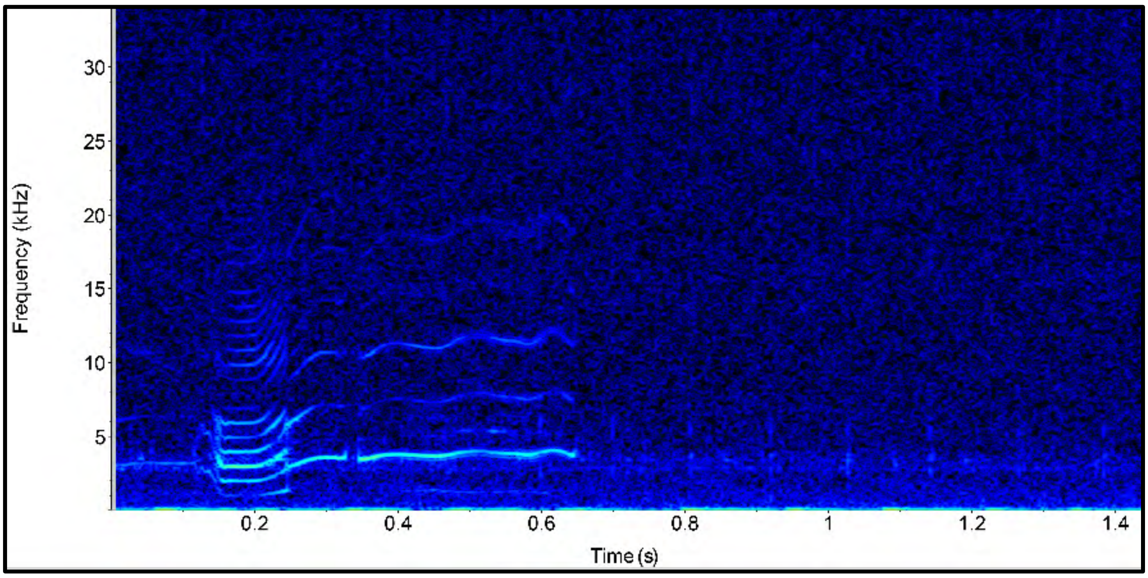

Call Type	Component	Statistic	Dur [s]	Fmin [Hz]	Fmax [Hz]	Fdelta [Hz]	BW 90% [Hz]	Fpeak [Hz]	Fcentre [Hz]	Q1F [Hz]	Q3F [Hz]	MinEnt [bits]	MaxEnt [bits]	AvgEnt [bits]	Dur90% [s]	Fstart [Hz]	Fend [Hz]	Ext	Infl	FM [1/s]	Steps
McM15a	1	Mean	0.16	622	21200	20578	4275	3384	3422	2934	3862	2.6	6.5	4.1	0.12						
n=54		*SD*	0.06	211	6329	6357	2131	308	301	451	476	0.5	0.8	0.5	0.04						
	2	Mean	0.35	1879	21000	19121	4144	3234	3183	3000	3956	1.8	6.9	3.1	0.27						
		SD	0.08	532	4795	4644	4465	865	551	444	968	0.5	0.5	0.8	0.07						
	Entire	Mean	0.51	599	22273	21674	4486	3422	3347	2873	3914	2.8	6.9	3.5	0.42						
		SD	0.09	229	5076	5109	2813	304	359	326	606	1.7	0.5	0.7	0.08						

Another example of Call Type McM15a:

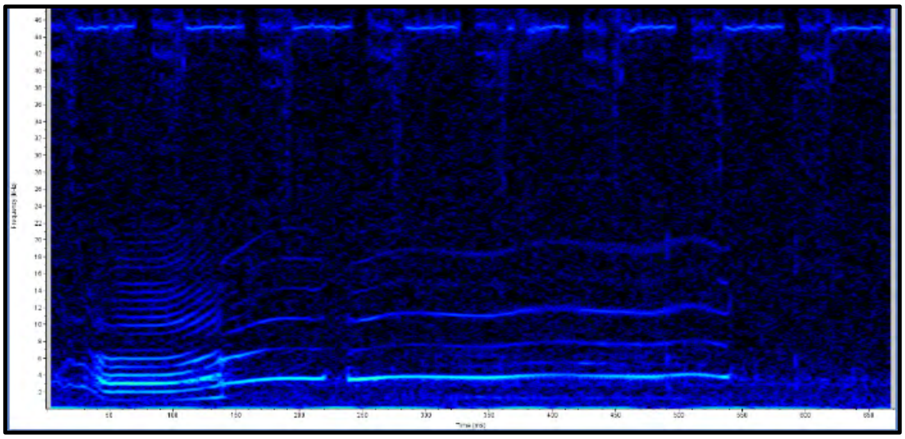

CALL TYPE McM16

This is a burst-pulse sound starting with a SBS of 1 kHz increasing to 2 kHz.

n = 7

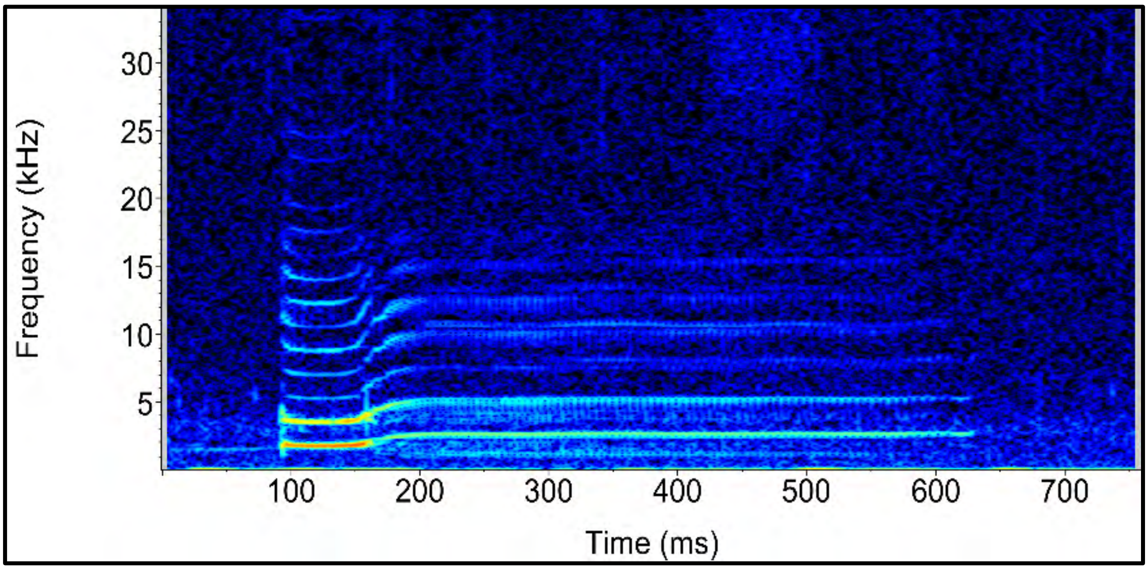

Call Type	Component	Statistic	Dur [s]	Fmin [Hz]	Fmax [Hz]	Fdelta [Hz]	BW 90% [Hz]	Fpeak [Hz]	Fcentre [Hz]	Q1F [Hz]	Q3F [Hz]	MinEnt [bits]	MaxEnt [bits]	AvgEnt [bits]	Dur90% [s]	Fstart [Hz]	Fend [Hz]	Ext	Infl	FM [1/s]	Steps
McM16	1	Mean	0.42	1193	15750	14557	2652	3134	2826	2451	3502	2.9	6.2	3.7	0.27						
n=7		*SD*	0.07	394	6030	5899	600	1291	855	890	841	1.6	0.4	0.9	0.10						

Another example of Call Type McM16:

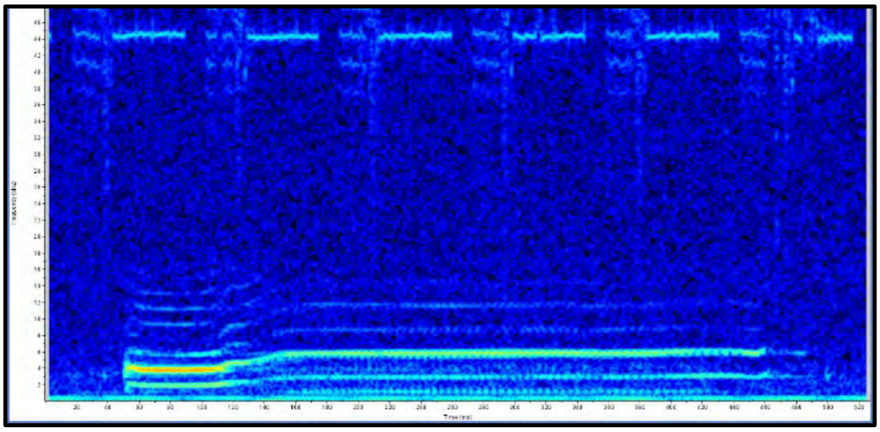

CALL TYPE McM17

This is a 2-component biphonic call consisting of a burst-pulse sound with an SBS of 1-2 kHz and a whistle with harmonics and high frequency-modulation.

n = 3

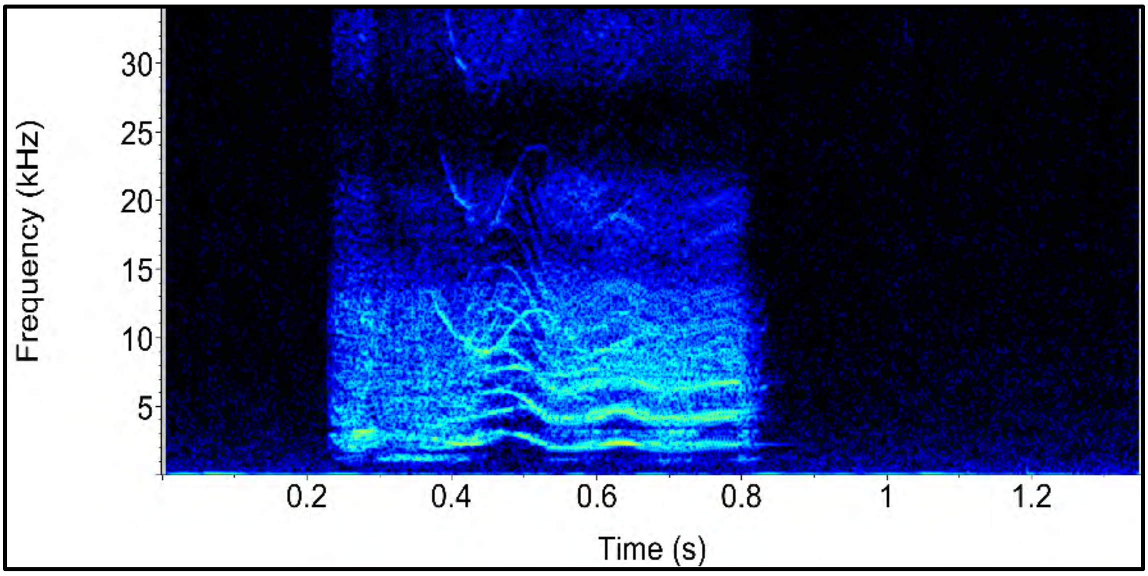

Call Type	Component	Statistic	Dur [s]	Fmin [Hz]	Fmax [Hz]	Fdelta [Hz]	BW 90% [Hz]	Fpeak [Hz]	Fcentre [Hz]	Q1F [Hz]	Q3F [Hz]	MinEnt [bits]	MaxEnt [bits]	AvgEnt [bits]	Dur90% [s]	Fstart [Hz]	Fend [Hz]	Ext	Infl	FM [1/s]	Steps
McM17	1	Mean	0.65	857	25578	24721	6078	2109	2844	2156	4359	3.6	6.2	4.3	0.50						
n=3		***SD***	0.12	166	1091	1251	3342	366	1382	430	2268	0.8	0.1	0.8	0.10						
	2	Mean	0.59	5540	13063	7523	3313	6016	6656	6125	7547	2.7	5.8	4.1	0.43	7977	8831	7	8	14.0	0
		SD	0.18	3289	1090	3069	985	3382	3851	3484	4357	0.2	0.5	0.5	0.06	6141	2165	3	3	1.1	0
	Entire	Mean	0.66	880	25615	24735	6078	2109	2844	2156	4359	3.5	6.2	4.3	0.50						
		SD	0.13	186	1078	1255	3342	366	1382	430	2268	0.8	0.1	0.8	0.10						

Another example of Call Type McM17:

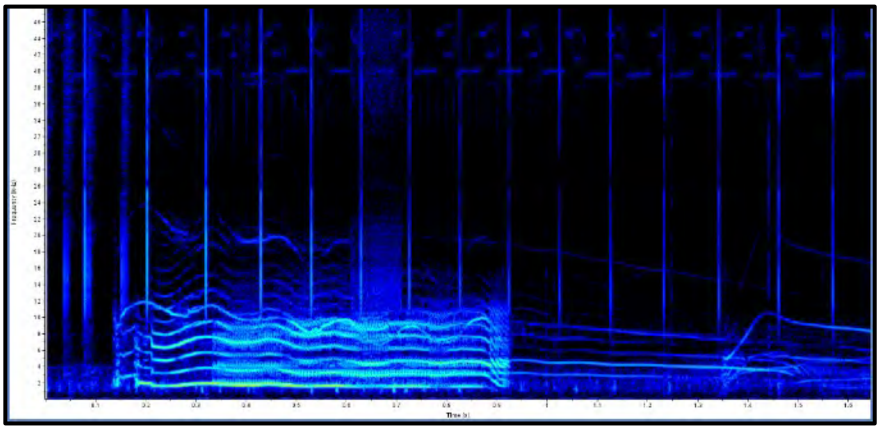

CALL TYPE McM18

This is a 3-component biphonic call. Component 1 consists of distinct pulses. Component 2 is a burst-pulse sound with variable SBS. Component 3 is a whistle with harmonics and high frequency-modulation. Components 2 and 3 make up the biphonation.

n = 3

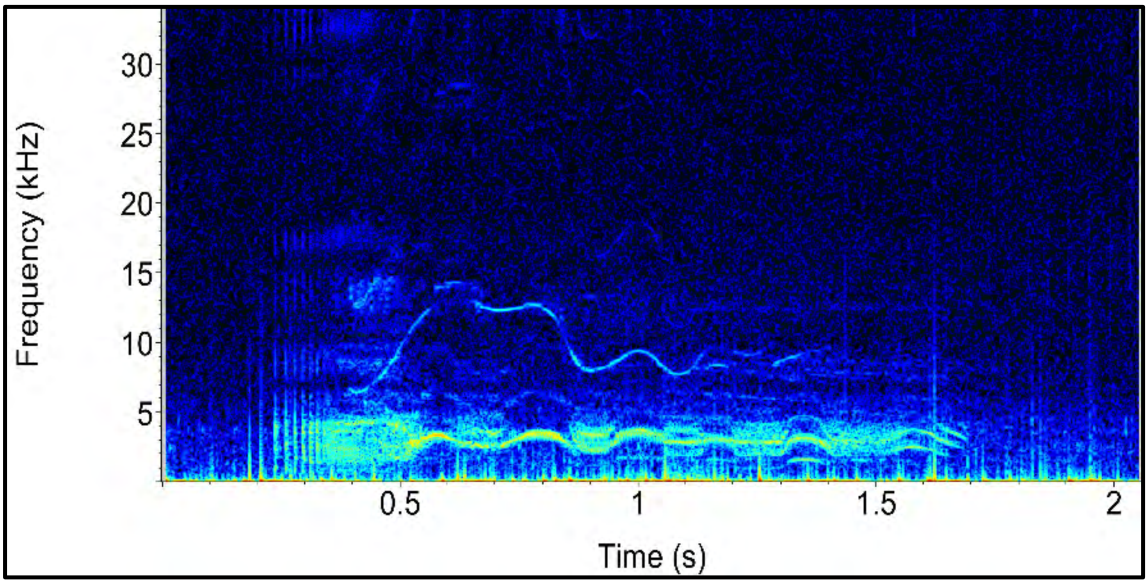

Call Type	Component	Statistic	Dur [s]	Fmin [Hz]	Fmax [Hz]	Fdelta [Hz]	BW 90% [Hz]	Fpeak [Hz]	Fcentre [Hz]	Q1F [Hz]	Q3F [Hz]	MinEnt [bits]	MaxEnt [bits]	AvgEnt [bits]	Dur90% [s]	Fstart [Hz]	Fend [Hz]	Ext	Infl	FM [1/s]	Steps
McM18	1	Mean	0.26	1821	29203	27382	9125	14359	16047	13969	18313	3.6	7.5	6.4	0.20						
n=3		*SD*	0.10	790	16317	15705	6100	19677	19374	19252	20831	0.5	1.5	1.7	0.10						
	2	Mean	1.22	1305	11467	10161	6875	4469	5125	2969	6313	3.8	5.9	4.6	1.00						
		SD	0.04	222	2361	2162	4441	2907	3357	871	3217	1.2	0.9	1.1	0.10						
	3	Mean	1.09	6163	12938	6775	5500	8516	9078	8313	10844	3.2	5.7	4.0	0.87	7050	9404	12	12	11.1	0
		SD	0.21	1275	523	1530	1597	683	404	423	1172	0.8	0.8	0.7	0.21	2351	801	3	4	3.6	0
	Entire	Mean	1.48	1305	29175	27870	16531	14234	14703	12531	17484	4.0	6.9	5.3	1.00						
		SD	0.06	247	16269	16091	18337	19789	19703	17321	20664	1.7	1.4	1.6	0.26						

Another example of Call Type McM18:

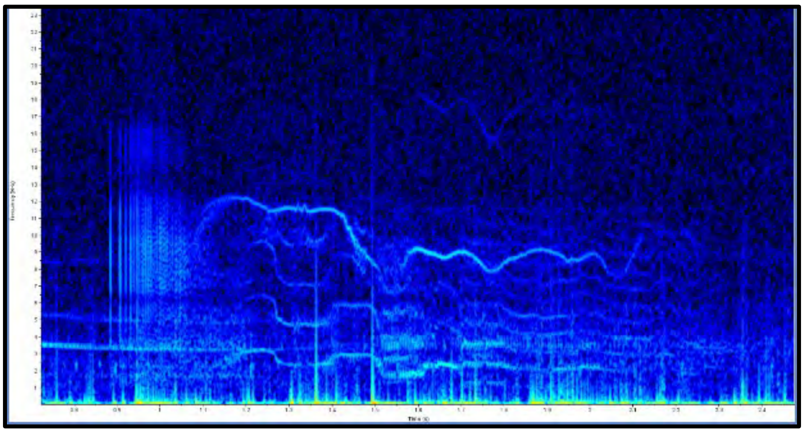

CALL TYPE McM19

This is a 3-component biphonic call. Component 1 is a series of distinct pulses increasing in PRR. Component 2 is a burst-pulse sound with a SBS of 2 kHz. Component 3 is a whistle with harmonics and AM. Components 2 and 3 form a biphonation. Note that the biphonic whistle commences near the end of component 1.

n = 4

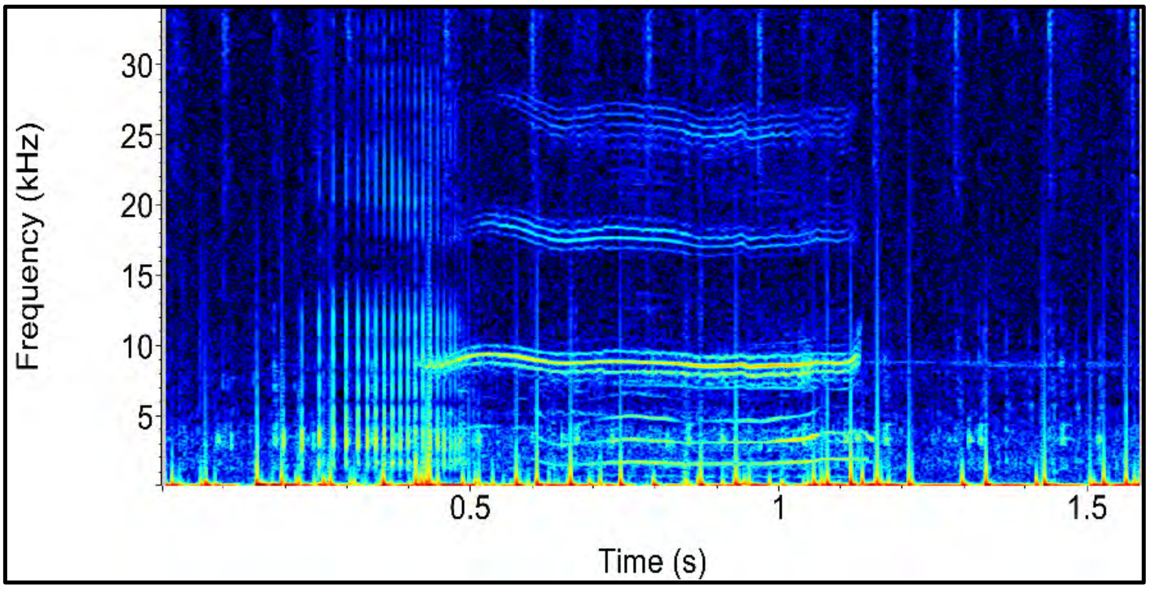

Call Type	Component	Statistic	Dur [s]	Fmin [Hz]	Fmax [Hz]	Fdelta [Hz]	BW 90% [Hz]	Fpeak [Hz]	Fcentre [Hz]	Q1F [Hz]	Q3F [Hz]	MinEnt [bits]	MaxEnt [bits]	AvgEnt [bits]	Dur90% [s]	Fstart [Hz]	Fend [Hz]	Ext	Infl	FM [1/s]	Steps
McM19	1	Mean	0.33	1668	36294	34626	19723	3820	5496	4184	9410	4.5	7.8	6.5	0.28						
n=4		*SD*	0.10	992	7897	8299	12658	4389	3067	2463	3355	1.0	1.0	0.9	0.05						
	2	Mean	0.93	1406	9125	7720	6668	4711	4629	3023	6152	2.9	5.4	4.2	0.63						
		SD	0.18	349	2138	1890	1174	3014	2996	1786	3062	1.0	0.5	0.8	0.15						
	3	Mean	0.78	7907	11747	3840	1523	8883	9141	8883	9633	2.1	4.9	2.5	0.58	9792	8635	3	4	4.5	0
		SD	0.14	1725	2072	2181	1472	835	532	811	673	0.7	1.2	0.9	0.10	3922	753	1	1	1.8	1
	Entire	Mean	1.24	830	36215	35385	11742	5496	7277	4242	9176	3.3	6.4	4.9	0.85						
		SD	0.23	492	7880	8171	4006	4161	2703	3239	1010	1.4	1.1	0.9	0.06						

Another example of Call Type McM19:

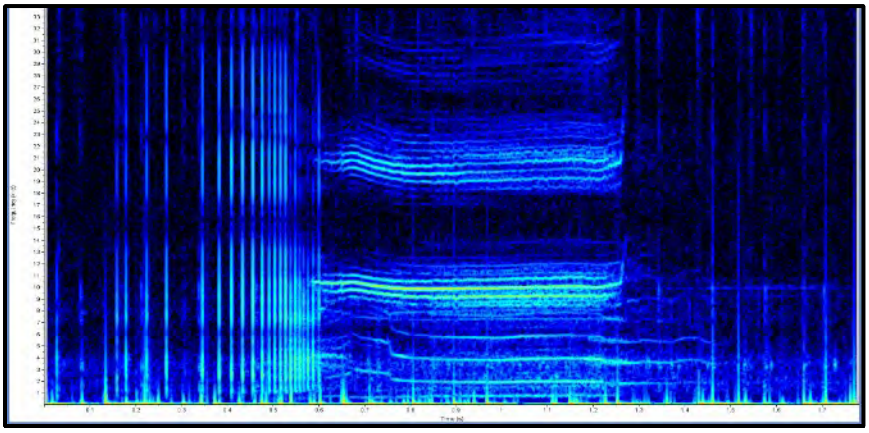

CALL TYPE McM20

This is a singular whistle with a long duration, low number of extrema and inflection points and hence low frequency-modulation rate.

n = 42

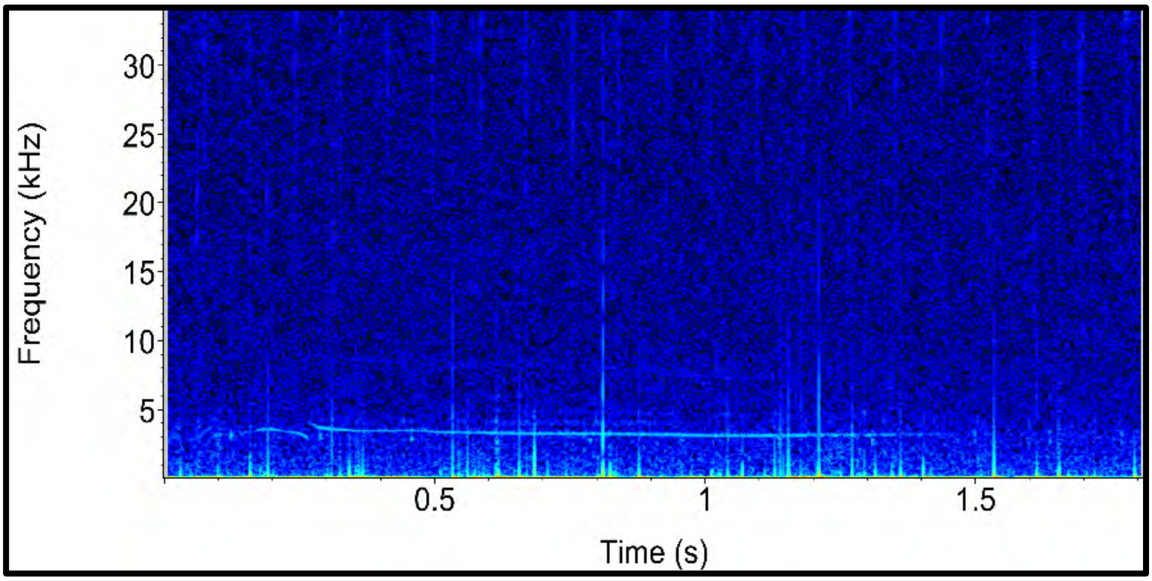

Call Type	Component	Statistic	Dur [s]	Fmin [Hz]	Fmax [Hz]	Fdelta [Hz]	BW 90% [Hz]	Fpeak [Hz]	Fcentre [Hz]	Q1F [Hz]	Q3F [Hz]	MinEnt [bits]	MaxEnt [bits]	AvgEnt [bits]	Dur90% [s]	Fstart [Hz]	Fend [Hz]	Ext	Infl	FM [1/s]	Steps
McM20	1	Mean	1.24	3863	6054	2190	1602	4534	4640	4372	5060	2.8	4.7	3.4	0.96	5166	4480	2	2	1.3	0
n=42		*SD*	0.60	2518	3587	1150	1065	2855	2909	2696	3207	0.8	0.9	1.1	0.49	3292	2681	3	3	1.9	0

Another example of Call Type McM20:

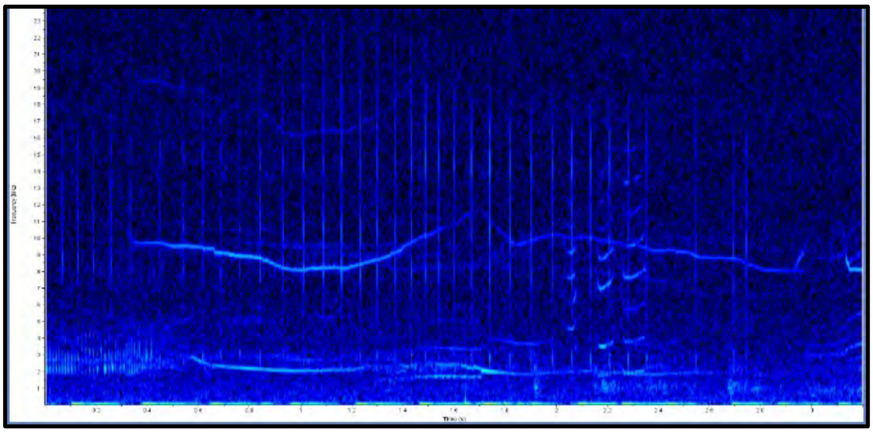

CALL TYPE McM21

This is a singular whistle with a short duration, low number of extrema and inflection points and hence low frequency-modulation rate.

n = 52

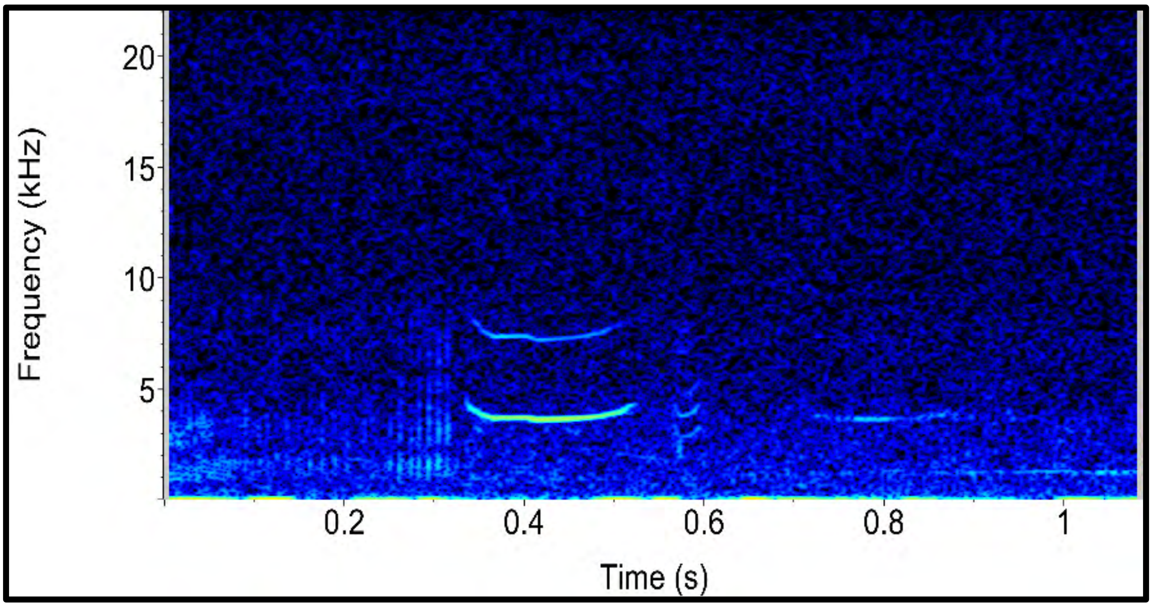

Call Type	Component	Statistic	Dur [s]	Fmin [Hz]	Fmax [Hz]	Fdelta [Hz]	BW 90% [Hz]	Fpeak [Hz]	Fcentre [Hz]	Q1F [Hz]	Q3F [Hz]	MinEnt [bits]	MaxEnt [bits]	AvgEnt [bits]	Dur90% [s]	Fstart [Hz]	Fend [Hz]	Ext	Infl	FM [1/s]	Steps
McM21	1	Mean	0.19	5456	7773	2317	1120	5830	5960	5829	6206	2.3	4.8	3.0	0.14	7459	6972	1	1	7.3	0
n=52		*SD*	0.08	3241	4166	1436	1056	3211	3261	3206	3398	0.6	1.0	0.8	0.08	4232	4044	1	0	7.5	0

Another example of Call Type McM21:

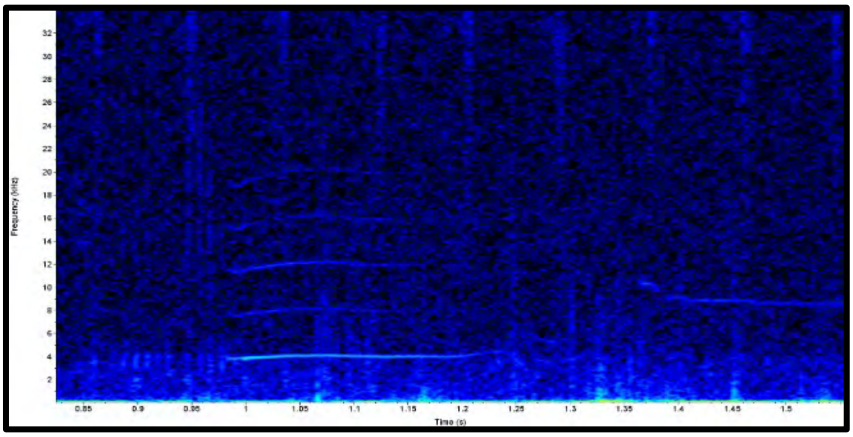

CALL TYPE McM22

This is a 4-component biphonic call. Component 1 is a burst-pulse sound, which sometimes starts as distinct pulses. Component 2 is a whistle with high frequency-modulation. Component 3 is another burst-pulse sound. Component 4 is a whistle with harmonics that commences as an upsweep and then decreases in frequency. The biphonic whistle starts during component 1 and ends during component 3.

n = 6

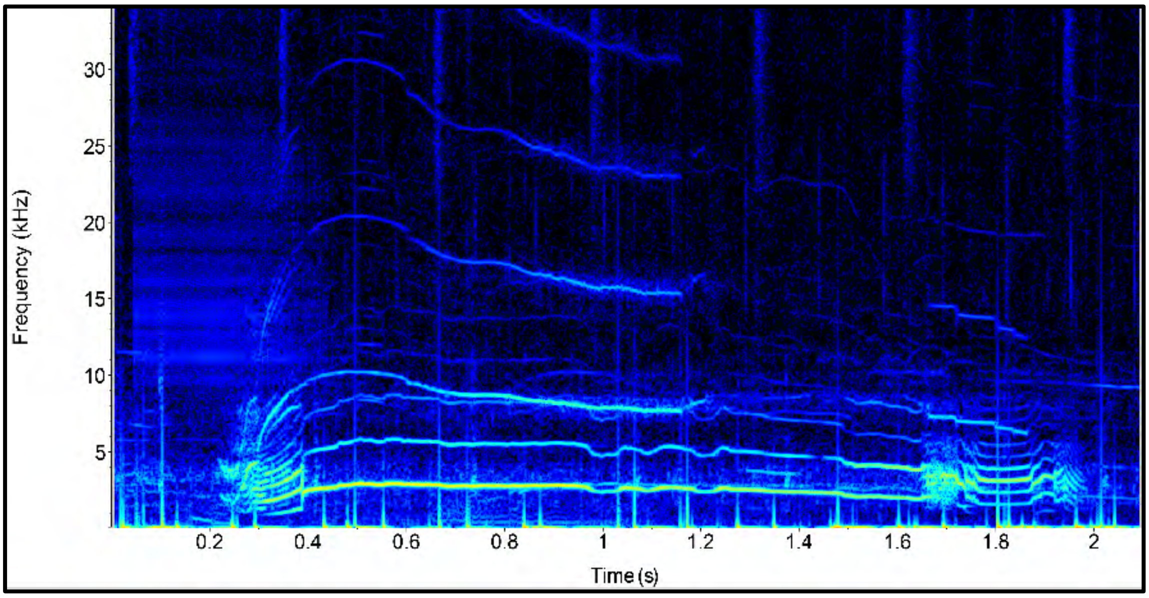

Call Type	Component	Statistic	Dur [s]	Fmin [Hz]	Fmax [Hz]	Fdelta [Hz]	BW 90% [Hz]	Fpeak [Hz]	Fcentre [Hz]	Q1F [Hz]	Q3F [Hz]	MinEnt [bits]	MaxEnt [bits]	AvgEnt [bits]	Dur90% [s]	Fstart [Hz]	Fend [Hz]	Ext	Infl	FM [1/s]	Steps
**McM22**	**1**	**Mean**	0.28	904	32617	31714	3906	2570	2805	2258	3336	3.7	6.9	5.1	0.20						
n=6		***SD***	0.10	411	16560	16402	1722	418	283	323	359	0.8	1.1	0.9	0.09						
	**2**	**Mean**	0.97	1284	3190	1906	953	2547	2687	2438	2898	1.6	3.8	2.1	0.75	2032	1339	8	9	10.9	0
		SD	0.41	233	447	535	343	378	348	275	361	0.6	0.7	0.6	0.34	464	311	4	4	5.0	0
	**3**	**Mean**	0.56	1175	11143	9967	3016	2648	2859	2508	3305	2.7	5.8	4.0	0.43						
		SD	0.32	349	5582	5484	1794	418	284	368	523	0.9	0.8	0.9	0.31						
	**4**	**Mean**	1.08	2978	10481	7502	3875	4219	4977	4188	6070	2.4	5.0	3.3	0.80	3067	8088	4	5	5.2	0
		SD	0.34	1407	1175	1859	2408	2303	2490	2262	3205	0.9	1.2	0.9	0.28	1469	1178	3	3	4.2	0
	**Entire**	**Mean**	1.81	890	32566	31675	4172	2625	2789	2516	3141	2.8	6.3	3.7	1.25						
		SD	0.64	412	15192	15066	2640	267	227	121	222	0.8	1.0	1.0	0.57						

Another example of Call Type McM22:

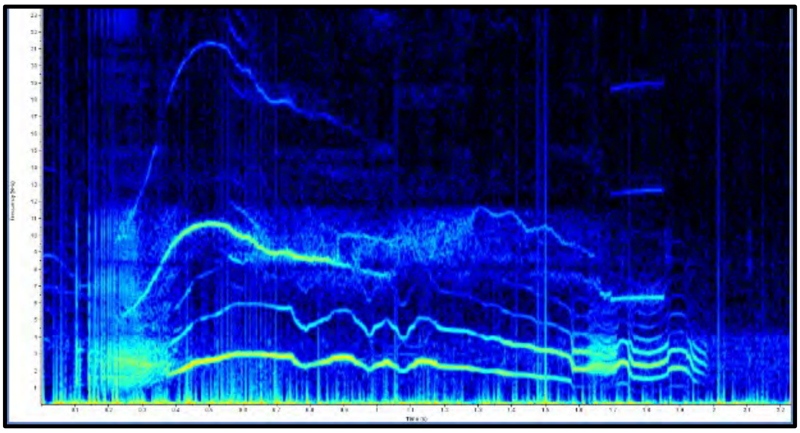

CALL TYPE McM23

This is a 2-component biphonic call. Component 1 is a burst-pulse sound with an SBS of 1-3 kHz. Component 2 is a whistle that starts as an upsweep but ends as a downsweep. Note that in the first image displayed here the burst-pulse sound from 0.5 to 0.9 s in the background is a call from a different animal.

n = 6

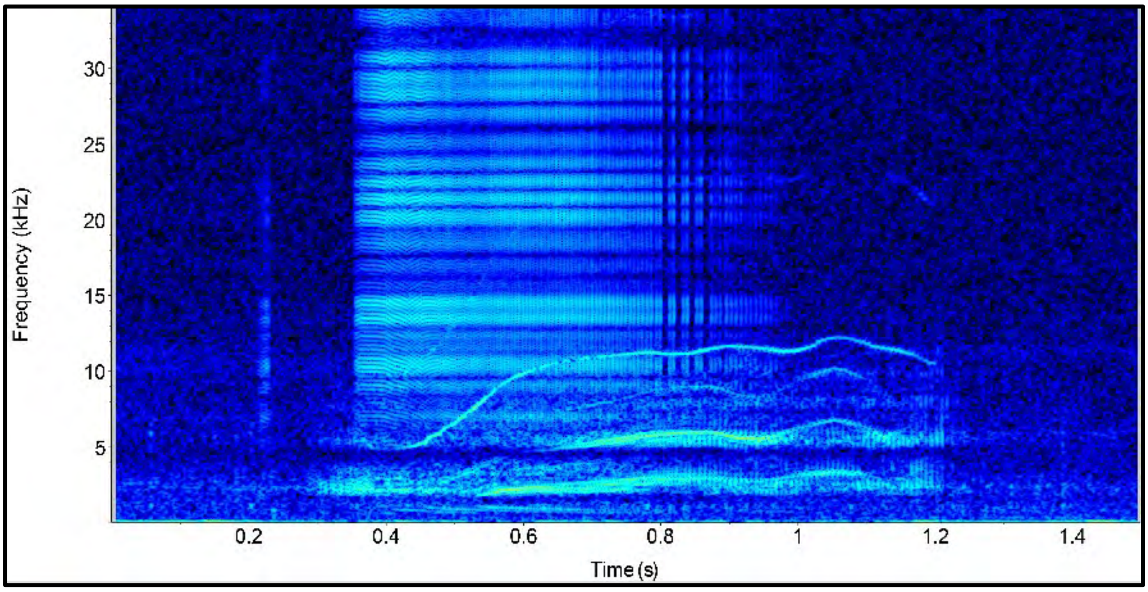

Call Type	Component	Statistic	Dur [s]	Fmin [Hz]	Fmax [Hz]	Fdelta [Hz]	BW 90% [Hz]	Fpeak [Hz]	Fcentre [Hz]	Q1F [Hz]	Q3F [Hz]	MinEnt [bits]	MaxEnt [bits]	AvgEnt [bits]	Dur90% [s]	Fstart [Hz]	Fend [Hz]	Ext	Infl	FM [1/s]	Steps
McM23	1	Mean	0.79	1528	11513	9985	7398	4578	5898	3859	7398	3.4	6.3	5.1	0.62						
n=6		*SD*	0.16	321	2403	2485	2130	2278	2659	1946	2466	0.5	0.3	0.5	0.13						
	2	Mean	0.79	4562	12581	8019	5930	6680	7047	6477	9062	2.9	5.9	4.8	0.62	4610	10515	7	8	9.6	0
		SD	0.15	1184	497	1030	1007	2018	1811	1711	1717	0.4	0.6	0.4	0.04	1253	2130	2	2	2.2	0
	Entire	Mean	0.82	1543	13276	11733	8406	4516	6008	3953	7641	3.6	6.4	5.3	0.65						
		SD	0.16	330	1613	1664	2127	2198	2580	2020	2345	0.9	0.3	0.5	0.10						

Another example of Call Type McM23:

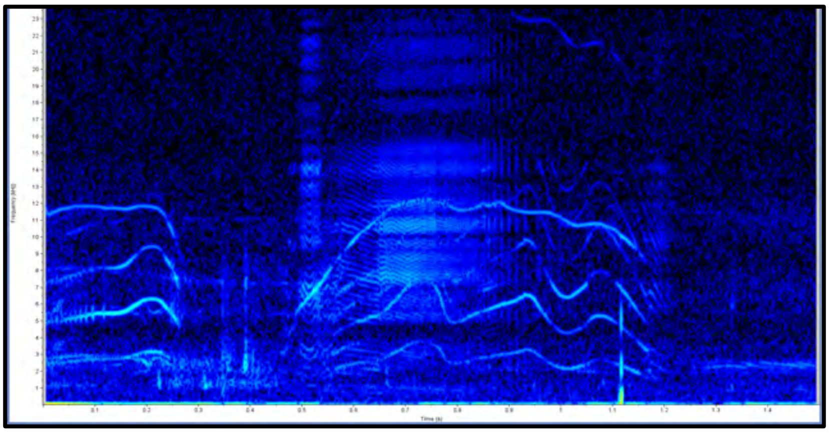

CALL TYPE McM24

This is a single component burst-pulse sound with SBS ranging from 500 Hz to 1 kHz.

n = 15

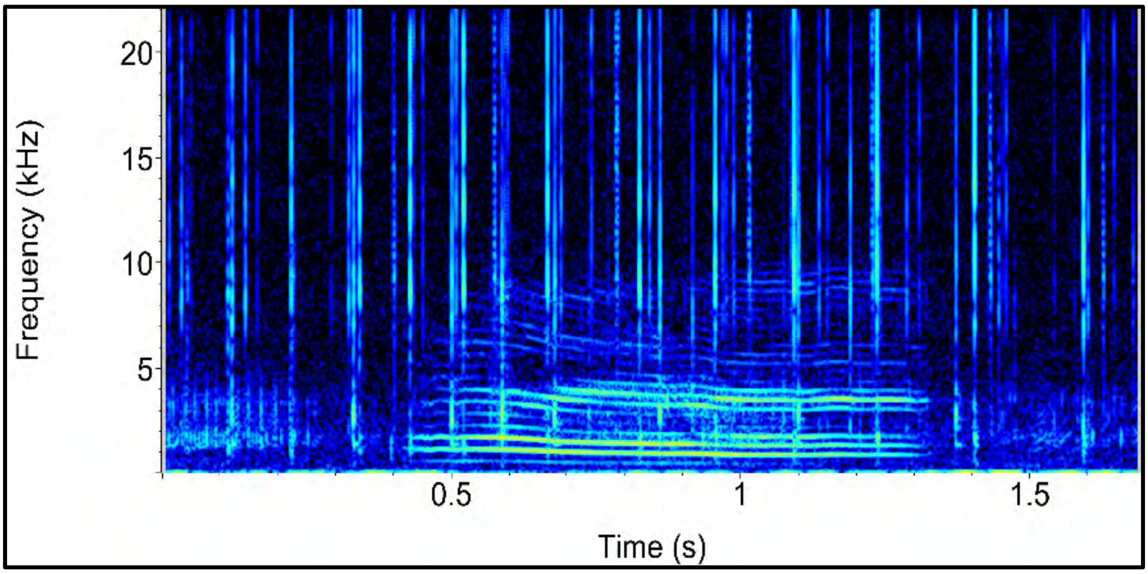

Call Type	Component	Statistic	Dur [s]	Fmin [Hz]	Fmax [Hz]	Fdelta [Hz]	BW 90% [Hz]	Fpeak [Hz]	Fcentre [Hz]	Q1F [Hz]	Q3F [Hz]	MinEnt [bits]	MaxEnt [bits]	AvgEnt [bits]	Dur90% [s]	Fstart [Hz]	Fend [Hz]	Ext	Infl	FM [1/s]	Steps
**McM24**	1	**Mean**	1.04	350	9552	9202	4422	1177	1450	1077	2383	2.9	6.6	4.5	0.77						
n=15		***SD***	0.33	149	2575	2713	3024	436	174	224	717	0.4	0.6	0.5	0.31						

<u>Another example of Call Type McM24:</u>

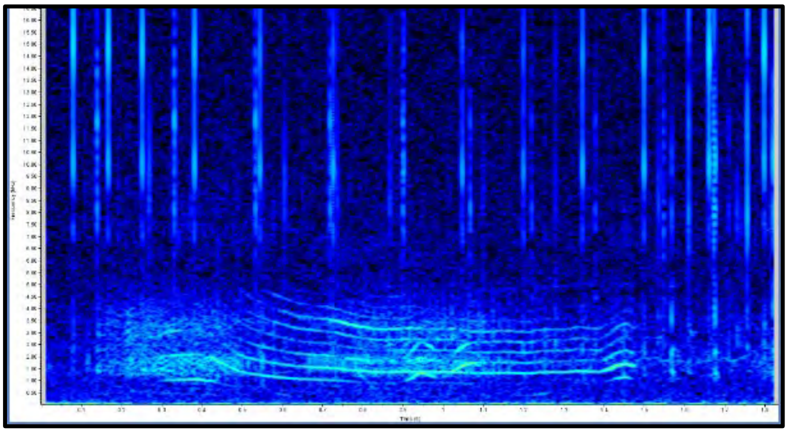

Supplement: Appendix 2: Type C Call Catalogue [file rsos191228supp2.pdf]
